# Great expectations, inconvenient truths, and the paradoxes of the dog-owner relationship for owners of brachycephalic dogs

**DOI:** 10.1371/journal.pone.0219918

**Published:** 2019-07-19

**Authors:** Rowena M. A. Packer, Dan G. O’Neill, Francesca Fletcher, Mark J. Farnworth

**Affiliations:** 1 Royal Veterinary College, Hawkshead Lane, North Mymms, Hertfordshire, United Kingdom; 2 Royal (Dick) School of Veterinary Studies, The University of Edinburgh, Easter Bush Veterinary Centre, Roslin, United Kingdom; 3 Animal Performance, Behaviour and Welfare Group, School of Animal Rural and Environmental Sciences, Nottingham Trent University, Nottingham, United Kingdom; Universidade do Porto Instituto de Biologia Molecular e Celular, PORTUGAL

## Abstract

Popularity of brachycephalic (flat-faced) dog breeds is increasing internationally despite well-documented intrinsic health and welfare problems associated with their conformation. Given this apparent paradox, greater understanding of the expectations and reality for brachycephalic dog owners and factors driving the dog-owner bond are needed. This study reports a large-scale online survey with valid responses from 2168 owners of brachycephalic dogs (Pugs: n = 789, median age of dogs 2.5 years; French Bulldog: n = 741, median age 2.0 years; Bulldogs: n = 638, median age 2.5 years). The most common owner-reported disorders in their dogs were allergies, corneal ulcers, skin fold infections and Brachycephalic Obstructive Airway Syndrome (BOAS). One-fifth (19.9%) of owners reported that their dog had undergone at least one conformation-related surgery, 36.5% of dogs were reported with a problem with heat regulation, and 17.9% with problems breathing. Despite awareness of their dog’s health issues, 70.9% owners considered their dog to be in very good health or the best health possible. Paradoxically, just 6.8% of owners considered their dog to be less healthy than average for their breed. Dog owner-relationships were extremely strong across all three breeds. Emotional closeness to their dog was highest for owners of Pugs, female owners, and owners with no children in the household. Ownership of brachycephalic dog breeds is a complex phenomenon, characterised by extremely strong dog-owner relationships and unrealistic perceptions of good health set against high levels of disease in relatively young dogs. Perceptual errors in owner beliefs appear to exist between brachycephalic owner perspectives of their own dog’s health versus the health of the rest of their breed, which may be fuelled by cognitive dissonance processes. These novel data improve our understanding of the cognitive processes and relationships that facilitate the rising popularity of breeds that paradoxically are affected by high levels of conformation-related morbidity.

## Introduction

There is increasing scientific evidence and international publicity surrounding the health challenges facing brachycephalic dog breeds [1]. Despite these activities, the popularity of some small to medium sized brachycephalic breeds such as the Pug, French Bulldog and English Bulldog has increased over the past decade both in the United Kingdom (UK) and internationally [2–4]. Brachycephalic breeds are strongly predisposed to a range of disorders intrinsically related to their conformation, including respiratory disease [5, 6], eye disease [7, 8], dystocia [9], spinal disease [10], heat stroke and pneumonia [11]. Brachycephalic breeds are reported with significantly shorter lifespans (median longevity: 8.6 years, interquartile range [IQR] 2.4–10.8) than moderate and non-brachycephalic dogs (median 12.7 years, IQR 11.1–15.0) [5]. In addition to disorders directly related to conformation, insurance data indicate that compared with non-brachycephalic dogs, brachycephalic dogs also experience a higher prevalence of health problems thought to be unrelated to conformation such as skin cancer [11]. Indeed, a 2013 survey of New Zealand-based veterinarians identified the Bulldog, Pug and French Bulldog as having “*health and welfare too compromised to continue breeding*” [12].

A variety of drivers have been identified that influence owner decision-making with regards to dog ownership. Physical health was considered an ‘extremely important’ trait of the ideal companion dog by 60.3% of Australian [13] and 62.7% of Italian dog owners [14]. However, owners of brachycephalic breeds have previously been demonstrated to be less influenced by breed health and longevity in breed selection compared with non-brachycephalic dog owners [15]. Indeed, studies of American Kennel Club registration statistics suggest that breed popularity was more associated with the physical appearance of dogs than with factors such as welfare-related breed characteristics (health, longevity), resulting in breeds with increased inherited disorders becoming more popular [16]. A similar association is also reflected from analyses of the past 28 years of Australian Kennel Club registration statistics, where breeds with a larger cephalic index (skull breadth to length ratio i.e. a flatter face) have become steadily more popular over time [3]. Several factors have been identified that drive the popularity of brachycephalic breeds, including beliefs about dog behavioural traits (e.g. brachycephalic breeds make good companion dogs and are good breeds for households with children) and conformational traits (breed size suited to lifestyle and appearance) [15]. The results of a study of Danish dog owners suggested that French Bulldog owners might represent ‘extrinsically motivated’ owners, i.e. those who acquire dogs to obtain status and attention from other people because of the distinctiveness or cuteness of their dog [17]. In addition, nearly one third of French Bulldog owners from that study planned to acquire a dog of the same breed in the future, more so than owners of any of the other small breeds studied (Chihuahua, Cairn Terrier, Cavalier King Charles Spaniel) [17].

The distinctive physical appearance of brachycephalic dogs’ faces, with their large foreheads, large and low‐lying eyes, and bulging cheeks, may also trigger instinctual human attraction to these breeds. These features are part of Lorenz’s ‘baby schema’, infantile facial stimuli that arouse positive emotions and nurturing responses in human adults [18]. Empirical studies have demonstrated that infantile features in cats, dogs and teddy bears increase their attractiveness, and that women show higher ratings for pets with infant features than men [19]. Thus, emotional drivers for ownership of brachycephalic breeds are complex, strong and multifactorial but nonetheless clearly highly influential in breed selection.

Much of the initial attraction from prospective owners towards brachycephalic breeds appears to focus on the ‘cute’ external appearance of these dogs; however, continued appeal during ownership, often in the face of chronic health disorders, suggests strong influences and misalignment of other elements of owner psychology. Unconscious, or indeed conscious, fallacies in owner perceptions of their dog’s true health status may perpetuate owner’s continuing satisfaction with their brachycephalic selection [20]. Over half of owners of brachycephalic dogs exhibiting clinical signs of BOAS did not recognise that their dog had a specific breathing ‘problem’, instead often justifying these signs as ‘normal for the breed’ [20]. As such, owner expectations of their dog’s health may be influenced by their choice of breed, with greater levels of morbidity tolerated by some breed owners. In addition, it is possible that owner perceptions of their dog’s health vary based on the strength of the bond that they have with that dog; a study of four small dog breeds in Denmark reported that French Bulldog owners had a stronger attachment to their dogs than Cairn Terrier owners [17]. The strength of the owner-pet bond has previously been identified as a factor influencing the level of veterinary care received by their pet [21]. Owners who exhibit strong bonds with their pets seek higher levels of veterinary care, are more likely to follow veterinarian recommendations regardless of cost, visit a veterinarian more frequently, and are more likely to seek preventive care [21]. As such, from these findings it might be expected that French Bulldog owners, and other brachycephalic dog owners (if strong dog-owner bondedness is a feature across breeds of this type) would be more attuned to their dog’s health status.

It is clear from the foregoing that deeper understanding is needed of the complexity of the beliefs and perceptions that brachycephalic dog owners hold on both their own dog and also their chosen breed in general. There is a critical need to appreciate the emotional and perceptual factors associated with the strength of bond between dog and owner if the paradoxical popularity of these health-challenged breeds is to be explained. To date, large scale studies of brachycephalic breed health have often focused on epidemiological methods from either first opinion or referral datasets with the aim of exploring quantitative aspects of brachycephalic dog health and to avoid potential biases introduced from owner reporting [22, 23]. However, this veterinary-centric approach is also limited by largely ignoring the perspectives and valuable insights of owners living with, and managing the daily husbandry of brachycephalic dogs. Careful analysis of these owner-related factors can greatly enhance the usefulness of the overall scientific evidence base on brachycephalic dog ownership and provide novel insights on why the continuing (or indeed, often increasing) popularity of these breed types persists in the face of rapidly expanding knowledge on their poor health. To start to fill these information gaps, this study aimed to:

1. Describe ownership experiences of brachycephalic dog owners, with special focus on:
  - Health events
  - Perception of their own dog’s health and the health of their chosen breed;
  - Expectations vs. realities of owning a brachycephalic dog
2. To compare 1(a)-(c) across three common brachycephalic breeds (French Bulldog, Pug and Bulldog) to explore breed-related differences in brachycephalic ownership experience
3. To explore the impact of 1(a)-(c) on the dog-owner bond in brachycephalic breeds

## Methods

### Participants

The three most commonly registered brachycephalic breeds with the Kennel Club (KC) are the French Bulldog, Pug and Bulldog [24]. Owners of these breeds were purposively sampled via breed-specific online forums and social media platforms (e.g. Twitter, Facebook) during June-July 2017. An explanatory letter was sent to the administrators of breed-related social media sites and a flyer containing a link to the questionnaire posted online once permission was granted. Participants were required to be 18 years of age or older and to currently own at least one of the three eligible breeds. Although distributed internationally, the questionnaire was only presented in English and thus a bias towards English speaking respondents was expected. Respondents were informed of the aims of the project and that submission of the survey would constitute their consent to the usage of these data for research purposes. Participants with more than one dog meeting the inclusion criteria were requested to answer the survey with regard to the most recently acquired dog. Recency effects have a strong influence on memory whereby more recent events are more readily recalled that older events [25], For this reason, dogs that were acquired more recently were more likely to have health events that were more recent and therefore owner recollection of such health events were likely to be less affected by recall bias [26]. The survey was granted approval by the Human Ethical Review Committee (HERC) at the R(D)SVS, University of Edinburgh.

### Questionnaire design

The questionnaire was designed iteratively amongst the authors with a small number of pilot respondents to ensure ease of understanding, and was hosted via Bristol Online Survey (www.onlinesurveys.ac.uk). Questions were grouped into six sections:

#### Section (1) Owner and dog demographics

Owners were asked to report: their own gender; age; number of children in the household; house type; income and education level; whether they were a first time dog owner. They were further asked to report their study dog’s age, sex, neuter status, breeding status, number of litters to date for female dogs, and age at acquisition.

#### Section (2) Veterinary history

Owners reported their dog’s veterinary costs to date (in £), which was used to calculate costs per year of ownership based on age at acquisition and current age. Owners reported which disorders had previously been diagnosed in their dog from a list of disorders affecting the airways, eyes, skin and spinal cord, and whether their dog had undergone conformation-altering surgery from a list common surgical treatments of these disorders. The disorder and surgery lists were generated from published clinical and epidemiological data on brachycephalic disease predisposition. Finally, owners of bitches were asked to report their dog’s whelping history, and any previous dystocia or caesarean section events.

#### Section (3) Airway dysfunction

Four health scores captured the severity of common brachycephaly-associated airway impairments: breathing difficulty, heat intolerance, eating difficulties and sleep dysfunction respectively. These health scores were either taken from previous studies [6, 20], or devised specifically for the current study. For breathing difficulty, the owner reported breathing score (ORB) captured both breathing difficulty and abnormal respiratory noise in a variety of situations, as a score out of 40 (8 and above being associated with clinically relevant airway obstruction) [20]. The following scoring systems were devised that combined scores for a number of sub-questions on heat intolerance (3 questions, scores: 0–12), eating difficulties (4 questions, scores: 0–20) and sleep dysfunction (6 questions, scores: 0–30) (Table 1).

**Table 1:** Owner reported scoring options for thermoregulatory, eating and sleeping dysfunction associated with brachycephaly.

**Thermo-regulatory score** **(Out of 12)**	**In warm weather, how often does your dog show the following behaviours?**										
	**Sign**		**Never**		**Rarely**		**Sometimes**		**Usually**		**Always**
	Heavy panting		0		1		2		3		4
	Laboured breathing		0		1		2		3		4
	Collapse		0		1		2		3		4
**Eating dysfunction score** **(Out of 20)**	**While or after eating, how often does your dog show the following behaviours?**										
	**Sign**	**Never**		**Rarely**		**Monthly**		**Weekly**		**Daily**	**> Once per day**
	Choking or gagging	0		1		2		3		4	5
	Laboured breathing	0		1		2		3		4	5
	Regurgitation	0		1		2		3		4	5
	Vomiting	0		1		2		3		4	5
**Sleep dysfunction score** **(Out of 30)**	**While sleeping, how often does your dog show the following behaviours?**										
	**Sign**	**Never**		**Rarely**		**Monthly**		**Weekly**		**Daily**	**> Once per day**
	Open mouth	0		1		2		3		4	5
	Sitting position	0		1		2		3		4	5
	Head in an elevated position	0		1		2		3		4	5
	With a toy in their mouth	0		1		2		3		4	5
	Stops breathing momentarily	0		1		2		3		4	5
	Changes positions frequently	0		1		2		3		4	5

#### Section (4) Owner perception of health problems

Owners reported whether they perceived (yes/no) their dog to have a ‘problem’ with breathing, heat regulation, eating or sleeping. Owners were then asked to score how they perceived their dog’s current health on a seven point ordinal scale from worst health possible, through to best health possible. Finally, owners reported how they perceived their own dog’s current health compared with their belief about the health of the rest of the same breed on a five point scale: much less healthy, less healthy, average, healthier than average, much healthier than average for the breed.

#### Section (5) Owner expectations

Owners reported how their expectations had been met or violated for four key areas of ownership: (1) veterinary costs, (2) exercise levels required by the dog, (3) maintenance levels and (4) overall behaviour. Areas 1–3 were scored as *less than expected*, *met expectations* or *more than expected*, and area 4 (behaviour) was scored as *worse than expected*, *met expectations*, or *better than expected*.

#### Section (6) Dog-owner bond

Owners completed the Monash Dog-Owner Relationship Scale (MDORS) which consists of 28 questions divided into three subscales: emotional closeness (EC; 10 questions), dog-owner interactions (DOI; 9 questions) and perceived costs (PC; 9 questions) [27]. Sub-scale EC focuses on the owner’s perception of the emotional support they derive from their dog and their bondedness with their dog. The DOI focuses on the frequency with which the owner engages with different activities e.g. playing with their dog, and PC focuses on how much owners feel inconvenienced or burdened by their dog. Questions for all three subscales are scored from 1–5 by the owner. What each number represented varied by question within each sub-scale, but most commonly, 1 = Never; 2 = Once a month; 3 = Once a week; 4 = Once every few days; 5 = At least once a day, or 1 = Strongly disagree; 2 = Disagree; 3 = Neither agree nor disagree; 4 = Agree; 5 = Strongly agree. For EC and DOI increasing scores represent rising quality of relationship (i.e. emotionally closer and engaging in more activities together, thus a more positive relationship); whereas for PC, increasing scores represent reducing quality of relationship (i.e. higher perceived costs of owning their dog indicate a less positive relationship).

### Statistical analyses

Following initial cleaning of data in Microsoft Excel 2013, statistical analyses were carried out in IBM SPSS Statistics v24 (SPSS Inc, Chicago, IL, USA). Univariable analyses used chi-squared tests for categorical*categorical variables (e.g. disorder diagnosis (0/1) * breed), Mann-Whitney U tests for non-normally distributed continuous*categorical data (e.g. MDORS score * breed) and Spearman’s rank for non-normally distributed continuous data (e.g. MDORS score * age of dog). Data distribution was ascertained by visual inspection of histograms. The three MDORS sub-scale scores were analysed as continuous variables. Linear regression was used to determine which factors predicted the three MDORS sub-scale scores. Twenty variables were tested for their association with the three MDORS sub-scale scores using separate linear regression models: canine demographics (breed, age); owner demographics (age, sex, whether they were a first time dog owner (1/0), children in household (1/0); veterinary experiences (vet costs per year (£); vet costs to date (£); number of conformation-related surgeries); health scores (ORB; heat intolerance score; eating difficulty score; sleeping dysfunction score) and owner perceptions of their dog’s health (number of perceived health problems; health compared to the rest of their dog’s breed; overall health rating); and owner expectations of the breed vs. reality of ownership (veterinary costs, exercise levels, overall behaviour, maintenance levels). Factors with liberal associations in univariable tests (*P* < 0.2) were taken forward for multivariable evaluation in generalised linear mixed models (glmms). Model development used backwards stepwise elimination and the Hosmer-Lemeshow test statistic was used to evaluate model fit. Country was included as a random effect in all models to take into account this potential source of non-independence, and its effects were assessed via its variance and changes in AIC value (with lower values indicating improved model fit). All biologically meaningful pairwise interactions were evaluated and retained, if significant. Final glmm model residuals were tested for model fit using the Hosmer-Lemeshow test and graphical analysis of residuals. Results are reported as mean ± standard deviation [SD] for normally distributed variables, and median [IQR] for non-normally distributed data. A p value of < 0.05 was considered significant.

## Results

### Section (1) Demographics

In total, 2168 valid responses were received from owners of Pugs (n = 789), French Bulldogs (n = 741) and Bulldogs (n = 638). Respondents were predominantly from the UK (72.0%) followed by USA (13.9%) and Canada (2.4%). The majority of respondents were aged 25–34 years (34.2%), with a minority ≤24 years (11.4%) or ≥55 years (9.0%). Respondents were predominantly female (89.1%). The highest level of education most commonly reached by respondents was an undergraduate degree (34.7%), and the most common household income was £25,000-£49,000 per annum (39.6%). Respondents most commonly lived in a detached (35.7%) or a semi-detached house (29.2%), and the majority had access to a garden (92.1%). Half of respondents lived in a suburban location (50.6%) followed by a rural location (26.4%) or an urban location (23.1%). Around half of households (45.7%) included children (demographics by breed: Table 2).

**Table 2:** Demographics of owners of Bulldogs, French Bulldogs and Pugs in the study population.

Variable	Sub-category	Bulldog N = 638	French Bulldog N = 741	Pug N = 789
Owner age (years)	18–24	7.5	12.7	13.3
	25–34	27.0	39.0	35.6
	35–44	34.6	26.9	21.8
	44–54	22.1	14.3	18.3
	55–64	7.4	5.1	8.9
	65–74	1.4	2.0	2.2
Owner gender	Female	85.7	89.6	91.2
	Male	13.9	10.3	8.2
Highest education level	GCSE	15.9	15.3	14.2
	Vocational qualification	27.1	20.0	24.6
	A Level	14.8	13.3	15.7
	Undergraduate degree	31.7	37.5	34.4
	Postgraduate degree	10.5	13.8	11.0
Household income	<£25,000	13.9	11.9	9.1
	£25,000 - £49,999	37.5	41.9	38.9
	£50,000 - £74,999	22.6	17.4	20.4
	£75,000 - £99,999	10.7	10.4	9.4
	> £100,000	13.9	11.9	9.1
Housing	Flat	9.4	18.9	11.2
	Terraced house	15.5	17.6	21.0
	Semi-detached house	29.8	28.2	29.7
	Detached house	40.5	32.2	35.2
	Other	4.7	3.1	2.9
Garden access	Yes	93.4	89.2	93.8
	No	6.6	10.8	6.2
Location	Rural	29.8	24.9	25.0
	Suburban	48.5	51.8	51.1
	Urban	21.7	23.3	23.9
Children in household	Yes	53.9	40.2	44.1
	No	46.1	59.8	55.9
First time owner	Yes	21.5	30.8	31.0
	No	78.5	69.2	69.0

The median age of study dogs was 2.17 years (0.92–4.33), with the majority of dogs aged under 5 years. In the study population, 58.4% of dogs were male and 42.0% of all dogs were entire. Of the male dogs, 89.7% had never been bred from and their owner had no intention to, 6.2% had not yet been bred from but their owner intended to, and 4.1% had sired at least one litter. Of the female dogs, 62.9% had never been bred from and their owner had no intention to, 6.4% had not yet been bred from but their owner intended to, and 30.7% had whelped at least one litter (1 litter = 23.4%, 2 litters = 5.6%, 3 litters = 1.1%, 4 litters = 0.6%) (data presented by breed: Table 3).

**Table 3:** Demographics of Bulldogs, French Bulldogs and Pugs in the study population.

Variable	Sub-category	Bulldog N = 638	French Bulldog N = 741	Pug N = 789
Age (years [IQR])	-	2.5 (1.0–5.0)	2.0 (1.0–3.5)	2.5 (1.0–5.0)
Sex	Female entire (%)	17.9	16.3	12.3
	Female neutered (%)	25.0	22.7	30.8
	Male entire (%)	28.6	30.4	21.7
	Male neutered (%)	28.6	30.7	35.2
Insured	Yes (%)	56.8	68.6	67.3
	No (%)	43.2	31.4	32.7
Breeding (Male)	1 or more litter(s) (%)	5.2	3.1	4.1
	Never—intend to (%)	6.9	7.3	4.5
	Never–do not intend to (%)	87.8	89.6	91.4
Breeding (Female)	1 or more litter(s) (%)	32.0	27.4	32.4
	Never—intend to (%)	7.0	9.4	3.3
	Never–do not intend to (%)	61.0	63.2	64.3

### Section (2) Veterinary history

#### Common diagnoses

The most common veterinary diagnoses from the options provided to owners were allergies (27.0%), corneal ulcers (15.4%), skin fold infections (15.0%) and BOAS (11.8%). The three breeds differed in prevalence for 7 of the 9 disorders assessed. Allergies and skin fold infections were most common in the Bulldog (33.7% and 22.4% respectively) whereas corneal ulcers were reported most commonly for the Pug (22.9%). BOAS did not substantially differ in owner-reported prevalence across the three breeds (Table 4). Bulldogs had the highest (or equal highest) prevalence for 5/9 conditions.

**Table 4:** Owner-reported diagnoses of common disorders in three brachycephalic breeds: Bulldogs, French Bulldogs and Pug (n = 2168). N.B. The p-value reports prevalence differences between the breeds.

Disorder type	Disorder	Overall %	Bulldog % N = 638	French Bulldog % N = 741	Pug % N = 789	p
Airway	BOAS	11.8	11.4	14.0	10.0	0.048
	Laryngeal collapse	5.1	5.3	5.3	4.7	0.826
	Tracheal hypoplasia	5.6	7.1	4.5	5.4	0.109
Ophthalmic	Corneal ulcer	15.4	14.6	8.1	22.9	<0.001
	Entropion and/or ectropion	9.5	19.1	3.9	6.8	<0.001
Spinal	Intervertebral disc disease	6.3	4.7	9.7	4.3	<0.001
	Spinal malformation	6.7	6.3	8.9	5.1	0.010
Skin	Skin fold infection	15.0	22.4	14.2	9.8	<0.001
	Allergies	27.0	33.7	28.9	19.8	<0.001

#### Conformation-related surgeries

Overall, 19.9% of owners reported that their dog had undergone one or more conformation-related surgeries. French Bulldogs had the highest prevalence for 4/8 conformation-related surgeries. The most frequently reported conformation-related surgeries were nostril widening (8.2%) and eyelid surgery (8.0%). The three breeds differed in prevalence for 4 of the 8 disorders conformation-related surgeries assessed. Nostril widening was most commonly reported in the French Bulldog (10.7%) and eyelid surgery in the Bulldog (18.0%) (Table 5).

**Table 5:** Owner-reported conformation-related surgeries in 3 brachycephalic breeds: Bulldogs, French Bulldogs and Pug (n = 2168). N.B. The p-value reports prevalence differences between the breeds.

Surgery	Overall %	Bulldog % N = 638	French Bulldog % N = 741	Pug % N = 789	p
Nostrils widening	8.2	5.5	10.7	8.1	0.002
Eyelid surgery	8.0	18.0	2.3	5.3	<0.001
Soft palate resection	7.6	6.4	9.3	7.0	0.092
Corneal surgery	4.2	4.4	2.0	6.1	0.001
Laryngeal saccule resection	2.8	1.6	3.6	2.9	0.061
Other skin fold reduction or removal	1.1	1.9	0.5	0.9	0.044
Nasal fold reduction or removal	0.7	0.3	0.8	1.0	0.295
Laser assisted turbinectomy	0.6	0.6	0.7	0.6	0.993

#### Dystocia and caesarean sections

Of the 275 female dogs that had previously been bred from, 61.1% were reported to have free-whelped all of their litters, while 38.9% required either medical or surgical intervention in at least one litter. Caesarean sections (without differentiation between elective and emergency surgeries) were more commonly reported in the Bulldog and French Bulldog than the Pug (p<0.001; Table 6).

**Table 6:** Owner-reported whelping experiences of 275 brachycephalic bitches.

Whelping experience (%)	Overall %	Bulldog % (n = 87)	French Bulldog % (n = 79)	Pug % (n = 109)
Free whelped (all litters)	60.7	48.3	51.9	78.0
Veterinary assistance with medication (one litter)	6.6	8.0	2.5	7.3
Veterinary assistance with medication (> one litter)	0.8	1.1	2.5	0.0
Caesarean section—elective or emergency (one litter)	9.8	27.6	31.6	10.1
Caesarean section—elective or emergency (> one litter)	5.7	14.9	11.4	4.6

#### Veterinary costs

**Fig 1:**
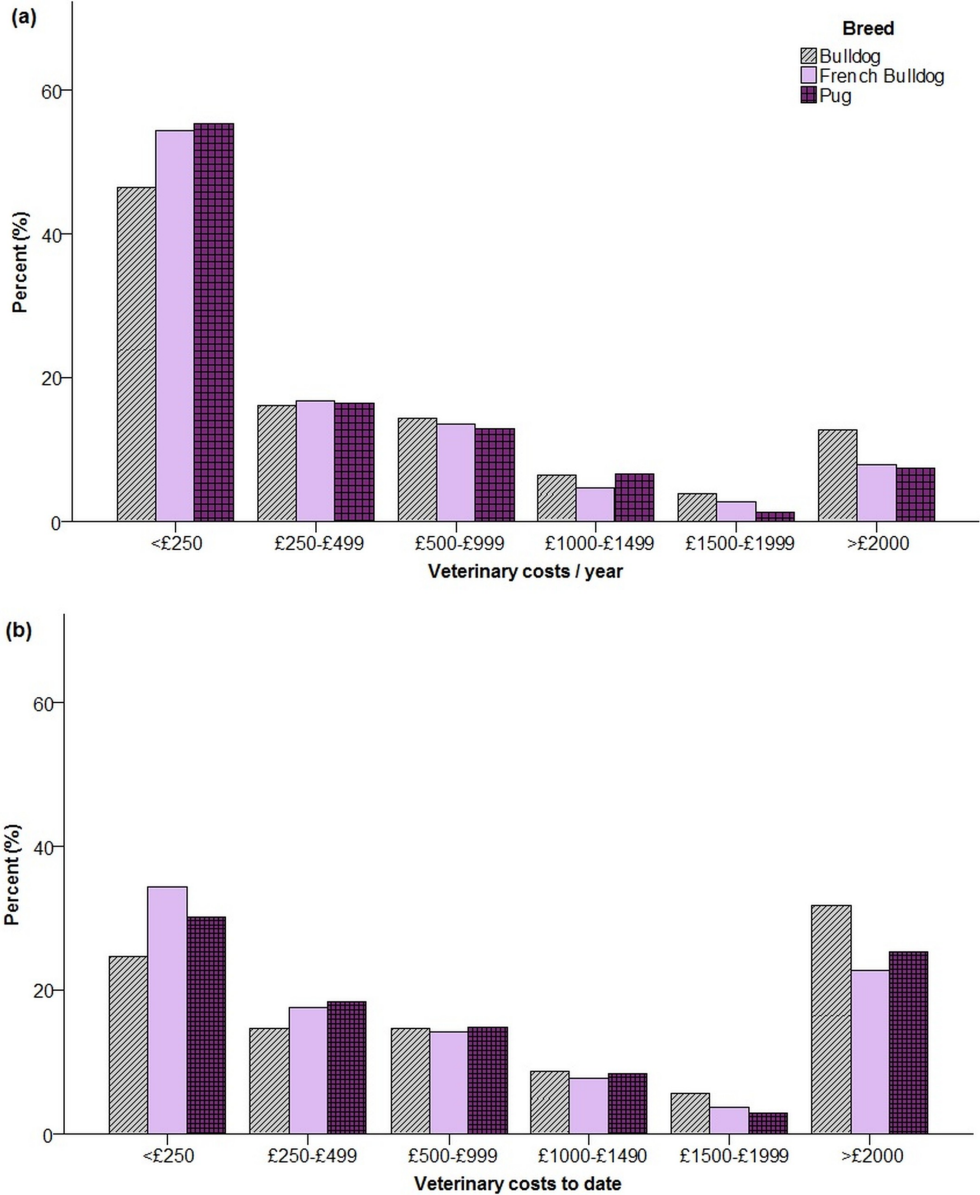
Veterinary costs (a) per year of ownership and (b) to date in 2168 brachycephalic dogs.

Across all three breeds, the median veterinary cost per year of ownership was £222.22 (IQR £90.90-£615.38), and median lifetime veterinary costs to date was £500.00 (£200.00-£2000.00). Bulldogs had the highest veterinary costs both per year and to date (Fig 1).

### Section (3) Airway impairments

**Fig 2:**
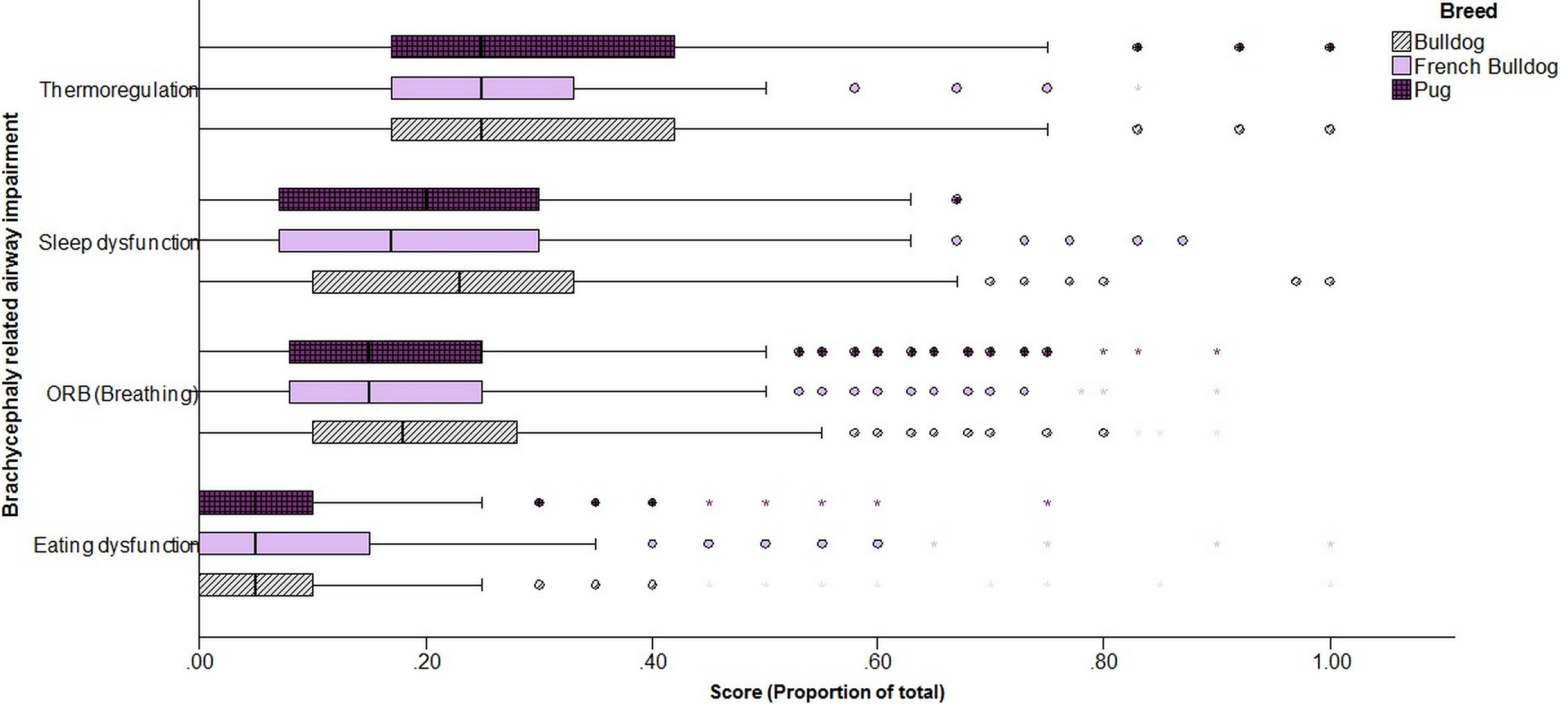
Severity of airway impairments in a population of Bulldogs, French Bulldogs and Pugs. N.B. Scores for each scale are expressed as a proportion of the maximum possible score to standardise across scales.

The median ORB score across all breeds was 6.0 out of 40 (IQR 3.0–11.0), with 39.1% (n = 847) receiving a score of 8 or above, indicating signs of clinically relevant airway disease [20]. Across the study population, the highest relative impairment score was for thermoregulation at 3.0 out of 12 (2.0–5.0) (Fig 2). Treating these scores as ordinal variables, ORB scores and sleep dysfunction scores were significantly higher in Bulldogs than French Bulldogs or Pugs (ORB: KW = 14.23, p<0.001; Sleep dysfunction: KW = 28.8, p<0.001), while scores related to difficulty eating were higher in French Bulldogs than Bulldogs or Pugs (KW = 21.33, p<0.001) (Fig 2).

### Section (4): Owner perception of health problems

**Fig 3:**
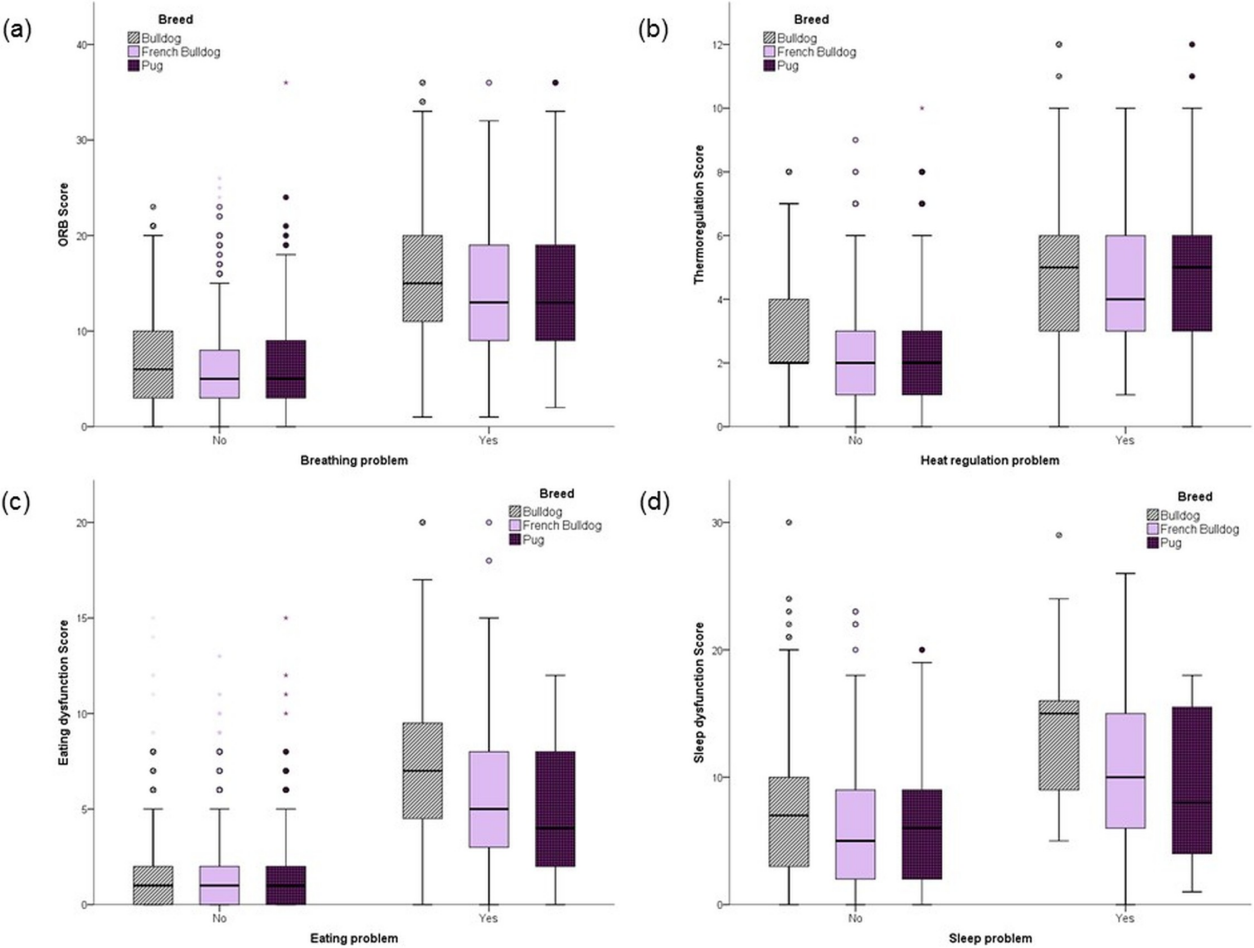
Health scores for 2168 brachycephalic dogs as reported by owners who do or do not perceive their dog to have a breathing, heat regulation, eating or sleeping problem. N.B. ORB Score = Owner Reported Breathing score. An ORB score of 8 or above is considered clinically relevant.

Owners were most likely to perceive that their dog had a problem with heat regulation (36.5%), followed by breathing (17.9%), eating (5.1%) and sleeping (2.7%). Bulldog owners were more likely to report a problem with eating than other breeds (7.2% vs. 4.1% French Bulldog, 4.2% Pug, p = 0.006), while French Bulldog owners were more likely to report a problem with sleeping than other breeds (3.9% vs. 2.4% Bulldog, 1.9% Pug, p = 0.043). Owners who perceived their dog to have a problem in any of these four areas also recorded correspondingly higher impairment scores (p<0.001) (Fig 3).

**Fig 4:**
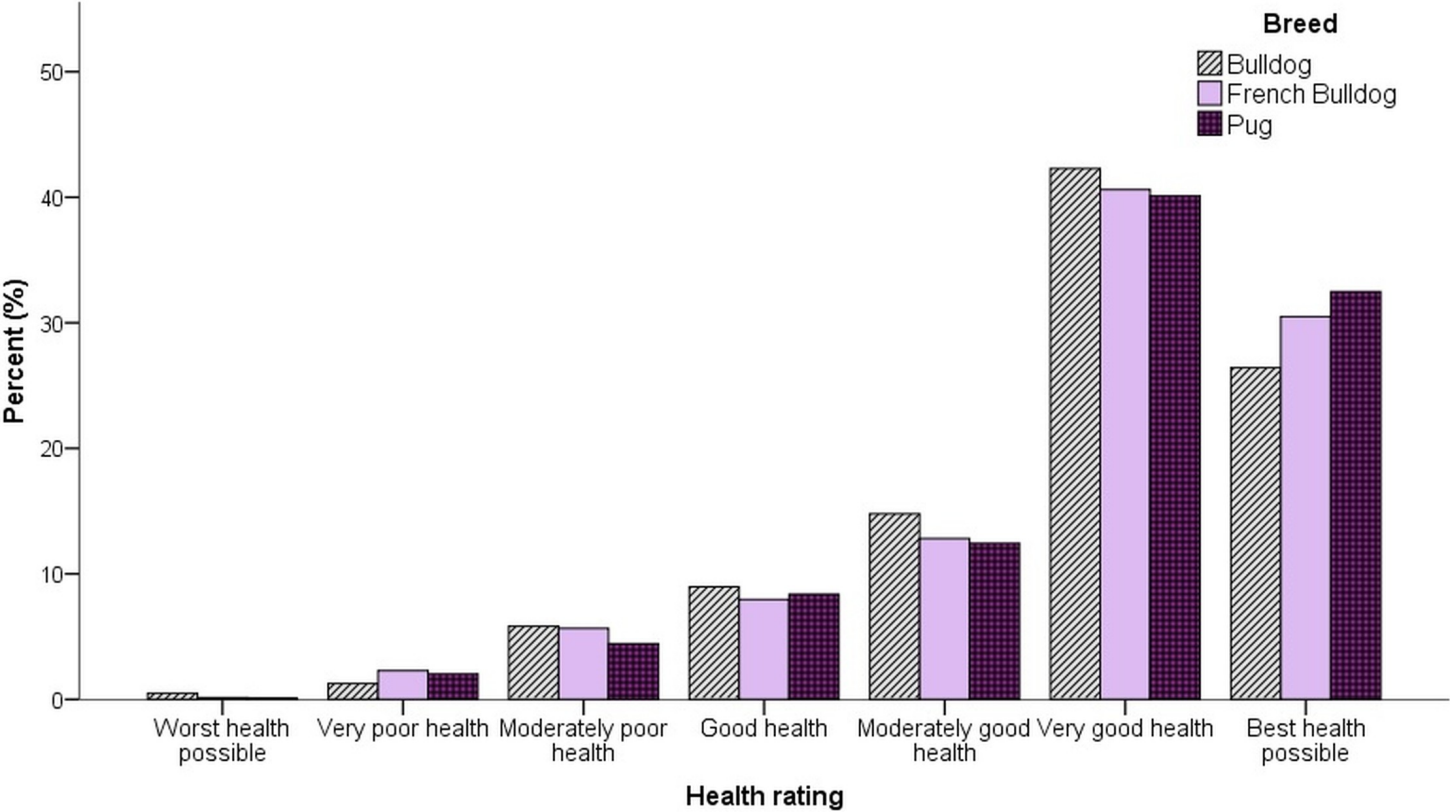
Owner ratings of their dog’s current health from the worst to best health possible.

**Fig 5:**
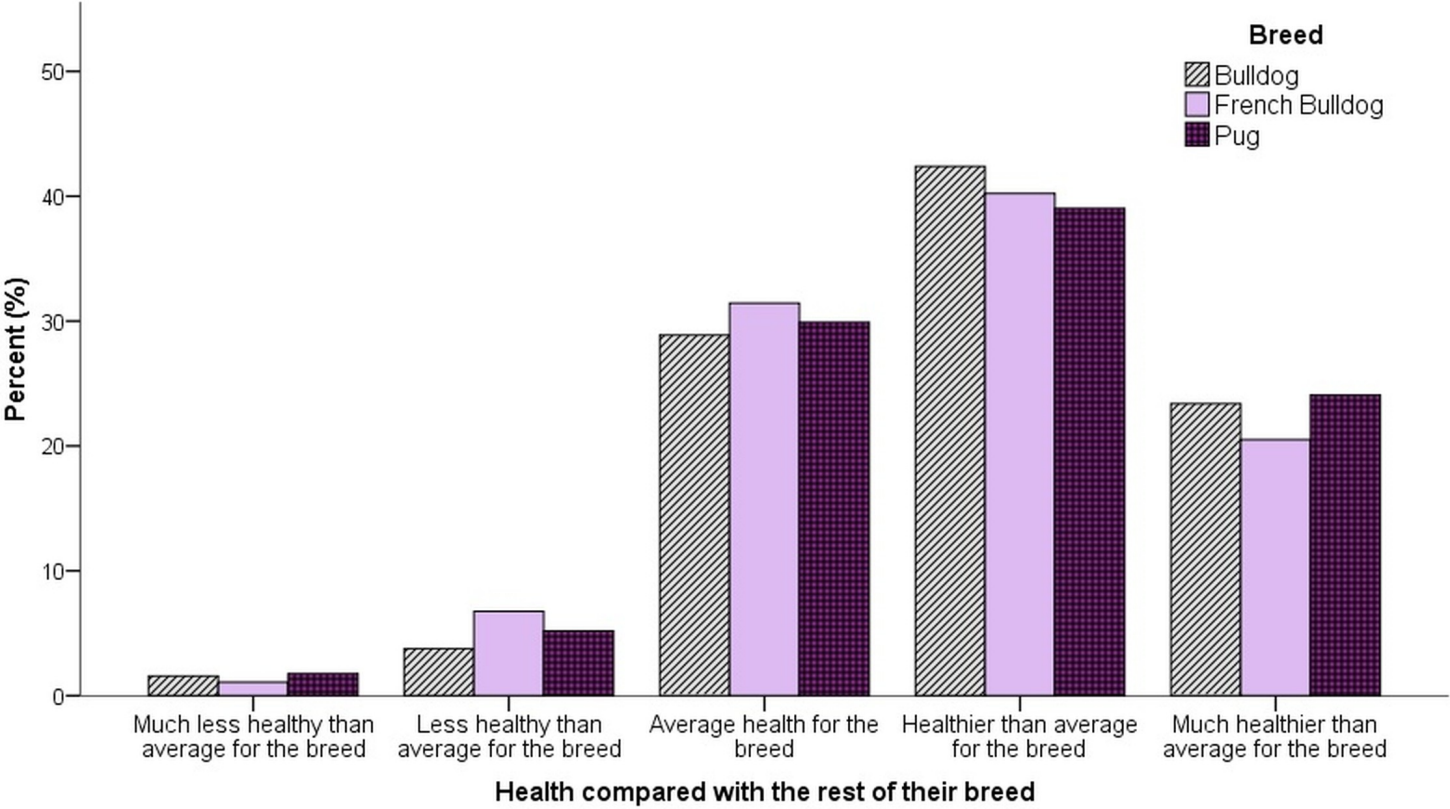
Owner ratings of their dog’s current health compared with the rest of their breed.

Overall, 70.9% of owners reported that they currently perceived that their dog was in either the ‘best health possible’ (30.0%) or ‘very good health’ (40.9%) (Fig 4). Compared with their perception of the health of the general population of their own dog’s breed, 63.1% of owners considered their own dog was either ‘much healthier than average’ (22.7%) or ‘healthier than average’ (40.4%) (Fig 5). Only 6.8% of owners considered their dog as either ‘less’ (5.3%) or ‘much less’ (1.5%) healthy than average for their breed.

### Section (5) Owner expectations of brachycephalic ownership

Expectations of owning a brachycephalic dog were violated for around one third of owners with regard to veterinary costs, exercise levels and their dog’s overall behaviour, and for one fifth of owners with regard to their maintenance levels (Table 7). One in five owners (21.7%) found their dog’s veterinary costs to be more than expected, while one in ten (9.4%) found their dog’s maintenance levels to be higher than expected. Bulldog owners were most likely to report their dog’s maintenance levels as being higher than expected compared to Pug and French Bulldog owners (Table 7).

**Table 7:** Owner expectations of health, behaviour and husbandry. N.B. The p-value reports differences between the breeds.

Variable	Subcategory	Overall	Bulldog N = 638	French Bulldog N = 741	Pug N = 789	P
Veterinary costs	Less than expected	13.0	15.3	11.5	12.5	0.093
	Met expectations	65.4	62.8	65.0	67.9	
	More than expected	21.7	21.9	23.5	19.7	
Exercise levels	Less than expected	9.9	11.7	9.6	8.8	0.137
	Met expectations	66.0	67.0	66.1	64.9	
	More than expected	24.1	21.3	24.3	26.3	
Overall behaviour expectations	Better than expected	21.0	21.2	21.1	20.8	0.678
	Met expectations	66.9	67.0	67.9	65.7	
	Worse than expected	12.1	11.8	11.0	13.5	
Maintenance levels	Less than expected	8.4	7.3	10.0	7.6	0.028
	Met expectations	82.2	81.2	82.9	82.5	
	More than expected	9.4	11.5	7.1	9.9	

### Section (6) Dog-owner bond

MDORS scores (1–5) were high for all three sub-scales with medians >4.0 for PEC, PC and DOI for all three breeds (Table 8). The final glmms for each sub-scale showed good fit (Hosmer-Lemeshow test: P>0.05) and normal distribution of residuals.

**Table 8:** Mean Monash Dog Owner Relationship Scale (MDORS) scores for the three MDORS subscales for Bulldogs, French Bulldogs and Bulldogs.

MDORS sub-scale (mean ± SD)	Bulldog N = 638	French Bulldog N = 741	Pug N = 789
Perceived emotional closeness (PEC)	4.32 ± 0.58	4.25 ± 0.60	4.33 ± 0.60
Perceived costs (PC) (n.b. higher score = lower costs)	4.20 ± 0.60	4.24 ± 0.52	4.31 ± 0.56
Dog owner interactions (DOI)	4.12 ± 0.45	4.13 ± 0.39	4.07 ± 0.43

**Fig 6:**
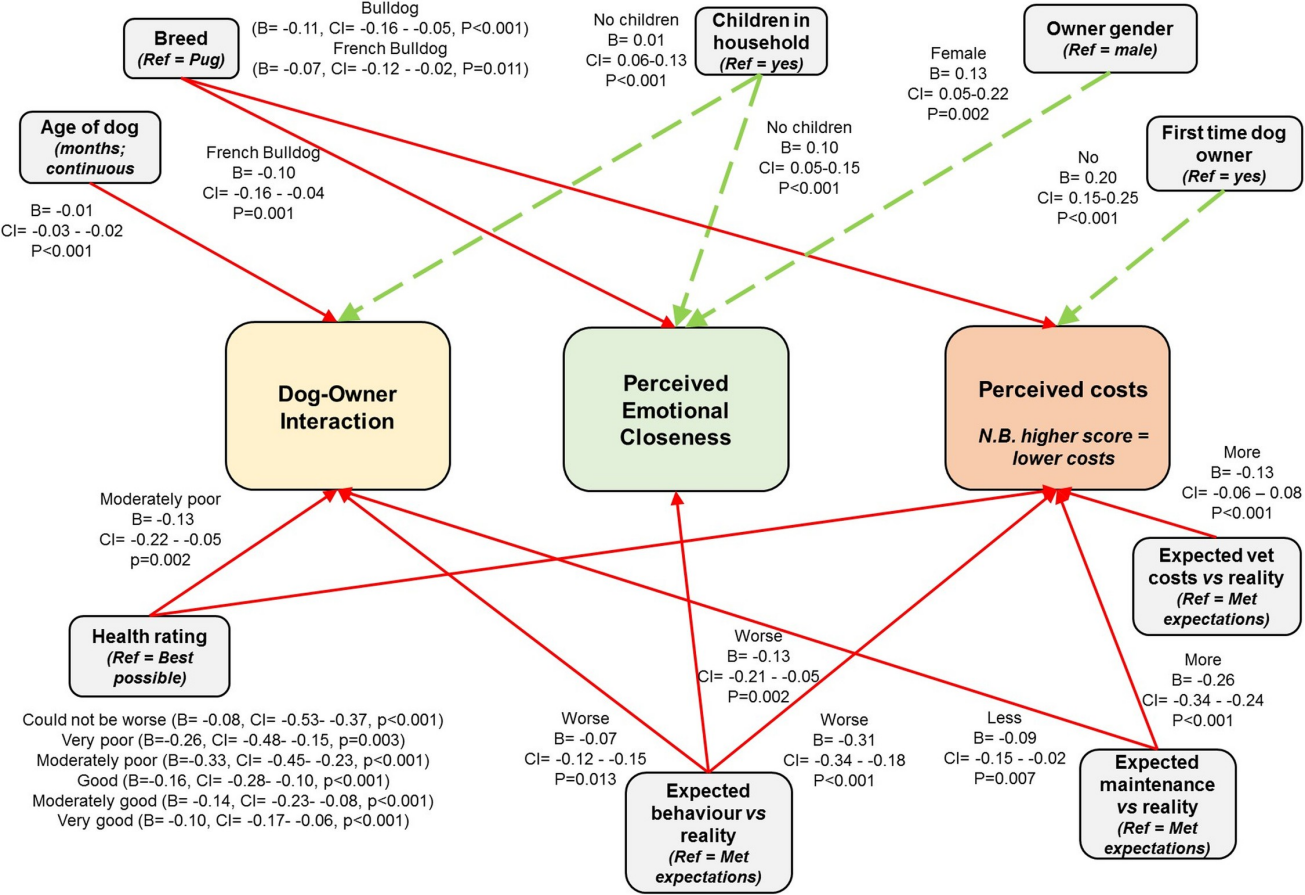
Schematic of factors influencing dog-owner bonding in brachycephalic breeds as quantified by three MDORS subscales.

Multivariable general linear mixed modelling identified four factors that were significantly associated with perceived emotional closeness (PEC), including owner and dog demographics and canine health (Fig 6). Pug owners exhibited higher PEC than French Bulldog owners; female owners exhibited higher PEC than male owners; and owners with no children in the household exhibited higher PEC than owners with children in the household. Owners whose dogs behaved worse than they expected exhibited lower PEC.

Six factors were associated with perceived costs (PC) of ownership; Bulldog and French Bulldog owners exhibited lower PC than Pug owners, and first time dog owners reported higher PC than previous dog owners. Owners of dogs with a worse health rating than the ‘best health possible’ reported higher PC. Owner expectations of owning their chosen breed influenced their perceived costs of ownership; owners whose dogs behaved worse than expected, experienced maintenance levels higher than expected or whose veterinary costs were more than expected reported higher PC.

Six factors were associated with dog-owner interactions (DOI); owners with no children in the household reported higher levels of DOI while owners with older dogs reported lower levels of DOI. Owners of dogs with moderately poor health reported lower levels of DOI compared to those with the ‘best health possible’. Owner expectations of owning their chosen breed also influenced their DOI, with owners of dogs that behaved worse than expected or had less maintenance than expected reporting lower levels of DOI.

Data are presented from three linear models, the outcome measure of each being one of the three subscales. Red arrows indicate a negative association and green arrows a positive association with the outcome variable. The reference category for categorical variables is stated in italics with the variable name; significant sub-categories are presented on the schematic. Country is included as a random effect in all models to take into account this potential source of non-independence.

## Discussion

This is the largest study to date to capture the ownership experiences, expectations and health perceptions of brachycephalic dog owners, with novel findings shedding light on the relationships that underpin the rising popularity of breeds that paradoxically are affected by high levels of conformation-related morbidity with reduced lifespan.

The owners in this study were predominantly young (mostly aged 25–34 years). This is in line with previous studies utilising online sampling that also reported that brachycephalic dog owners are younger than non-brachycephalic dog owners and speculated that this could be due to this younger age group being more susceptible to social influence that promotes the purchase of popular breeds [15]. Additionally, high levels of brachycephalic-owning households in the current study contained children (40–54%) so breed selection may also be influenced by perceptions that these breeds are good with children [15]. Despite the high levels of disease reported, the dogs in this study were generally young (median age of 2.17 years) as expected for breeds that are rapidly growing in popularity and where the overwhelming proportion of new puppy entrants move the median age of the population downwards. It is likely that the prevalence, spectrum and severity of disorders in these dogs will increase as this currently young population ages [28, 29]. This suggests that even the alarmingly high disease prevalence values reported in the current study may still be an underestimate of the true age-standardised disease prevalence that will be shown by the study dogs over time. It may be that this deteriorating health may affect owner perceptions of disease and weaken the dog-owner bond over time, and as such, future longitudinal studies of dog-owner dyads could supplement the cross-sectional approaches taken in the current study.

### Health experiences

As discussed above, despite the relatively youthful age of the study population, there was significant morbidity reported across the three breeds. Although this study did not intend to report reliable prevalence estimates of the conditions studied due to the inherent biases of sampling using an online convenience method, much of the health data presented concur with the current gold-standard for collecting such data, VetCompass. VetCompass holds data for nearly 10 million animals from 1,100 UK participating veterinary practices (over 20% of all UK practices). Although the data presented in this study are largely from the UK, over one quarter (28%) of dogs in this sample were from other countries. As reliable prevalence data on the conditions discussed are not yet available from similar systems outside of the UK, VetCompass studies are used for key health comparisons here.

Of the disease list assessed, allergies were the most commonly reported disorder in the study population, with over one quarter of all dogs diagnosed, rising to one third for the Bulldog. These data concur with Vetcompass studies of brachycephalic breed health, where skin disorders were the most common group-level disorder in the French Bulldog and Bulldog [28, 30], and second most common in the Pug [29]. In addition to allergic skin disease, skin fold infections were the third most commonly reported disorder in this study at 15.5% overall, but affecting almost one quarter of Bulldogs (22.4%). This owner-reported study prevalence is markedly higher than that of a recent VetCompass study that used first opinion veterinary clinical data to show a 7.8% prevalence [30]. This three-fold difference may be partially explained by selection bias in the current (online) study population towards owners of dogs with disease. The study used a convenience sample, as at present the lack of compulsory register for owners of dogs in most countries internationally makes random sampling, representative of the general dog owning population challenging or impossible. This difference may also be explained by veterinary misclassification bias whereby skin fold disease is under-diagnosed in the first opinion population, or differences in sampling frame between these two studies; owners in the present study were asked to report all diagnoses to date (lifetime disorder prevalence), whereas VetCompass studies focus on diagnoses solely within a single year (one-year disorder prevalence). Either way, it is clear that skin fold disease is a common and important health concern, especially in the Bulldog.

Corneal ulceration was the second most commonly reported disorder in this study, affecting 15.1% of dogs overall but nearly one quarter (22.9%) of Pugs. Corneal ulcers have been reported as the second most common disorder in Pugs with a prevalence of 8.7% [29]. This breed predisposition has been suggested to result from their high-risk craniofacial conformation including an extremely flattened face, wide eyes and nasal fold [7, 8]. Finally, the fourth most common disorder in the study population was BOAS, with a prevalence of 11.8% and no difference across the three breeds. Upper respiratory tract disease has been reported in 22% of extreme brachycephalic breeds [31], and is associated with their foreshortened muzzle and thick neck girth [6].

High proportions of owners had bred from their bitches in the current study, with around one third of all bitches having had at least one litter to date. Of these bitches, over one third required medical or surgical intervention to give birth in at least one litter. Caesearean surgeries were most common in Bulldogs (42.5%) and French Bulldogs (43.0%), compared to Pugs (14.7%). Pugs and French Bulldogs have previously been identified as predisposed to requiring caesarean sections to give birth [32], thought to (in part) be due to feto-pelvic disproportion associated with selection for relatively large heads and narrow hips in these breeds. Since 2012, the Kennel Club will not register puppies born by caesarean section from a bitch that has previously had two such surgeries [33]. Consequently, owners may be disinclined to report to the Kennel Club that their bitch had undergone such procedures, highlighting the importance that veterinarians should report caesarean sections that they perform to the Kennel Club [34]. Similarly, veterinarians are urged to also report conformation-altering surgeries that they perform [34]; indeed, in the current study, one fifth of dogs had undergone one or more conformation-related surgeries, the most common being nostril widening.

### Airway impairments and owner recognition of these problems in brachycephalic dogs

Impacts from partially or fully obstructed airways over a prolonged period are diverse and often highly detrimental to welfare, but studies to date often exclusively focus on breathing difficulties while awake. The current study presents concerning results suggesting that their compromised airways are leading to severely reduced physiological function and ability to cope with the everyday challenges of regular pet dogs. The ability to thermoregulate had the highest impairment score for these three brachycephalic breeds. Over one third of owners perceived that their dog struggled to regulate their body heat and core temperature. In agreement, experimental studies have confirmed that brachycephalic dogs that are exposed to higher temperatures have a decreased capacity for thermoregulation compared with non-brachycephalic dogs [35], and in the real world, a case series of heat stroke in dogs showed that brachycephalic breeds were overrepresented [36].To avoid such situations, owners of vulnerable breeds need to design, modify or avoid husbandry practices that may put dogs at unacceptable risk of overheating e.g. walking in hot temperatures, obesity, limited access to ventilation or shade [37].

Based on owner replies to clinical questions, nearly 40% of dogs reached an ORB score indicative of clinically relevant airway obstruction [20]. In contrast, when asked directly, only 17.9% of owners of these dogs considered their dog to have a breathing problem. These contrasting and paradoxical results support the influence here of the ‘normalisation’ phenomenon whereby owners of brachycephalic dogs may be consciously aware that the dog is struggling to breathe but not consciously accept that this is a specific problem, instead considering it a ‘normal’ and therefore somehow acceptable feature of the breed [6]. In addition to wakeful breathing dysfunction, sleep dysfunction scored as the second highest area of airway impairment in these three breeds, with the Bulldog most severely affected; the Bulldog breed has previously studied as a naturally occurring model of sleep apnoea [38]. However, just 2.7% of owners reported their dog to have a sleeping problem. This contrasts with a previous study of 100 brachycephalic dogs (Pugs and French Bulldogs) referred for the surgical treatment of BOAS (whose owners presumably recognised their dog had a breathing problem), in which 50% of owners reported sleep problems [39]. It is possible that the current study population were markedly less affected by sleep problems; however, it is also likely that many owners do not recognise sleep problems as a welfare issue [40] and may instead interpret signs of sleep-related airway impairment as benign ‘normal’ phenomena. For example, sleeping with a toy in their mouth or in a sitting position (strategies to avoid upper airway obstruction) may be considered as just cute quirks of their dog rather than indicators of true pathology. Finally, eating dysfunction received the lowest score of the four issues assessed, with just 5.1% of owners reported an eating problem in their dog. This again contrasts with a previous study of dogs with BOAS, in which 46% were reported with eating problems [39]. Both of these studies identified breed differences, with more frequent gastrointestinal signs associated with eating in French Bulldogs than the other breeds. GI signs in these breeds are often associated with gastrointestinal tract abnormalities including oesophageal, gastric and duodenal anomalies [41]. These issues may be directly associated with brachycephaly, breed-specific anatomical issues, or may be a combination of both.

For all four areas of dysfunction, greater severity of signs was significantly associated with recognition of a problem in that domain, indicating that dogs may need to reach a critical level of clinical severity (a threshold) before owners consciously acknowledge their dog has a ‘problem’ that is somehow worse than just being ‘normal for the breed’. These normalisation and thresholding phenomena are of welfare concern because they may prolong suffering and worsen prognosis in affected dogs. For example, owners may delay seeking veterinary advice and/or intervention until secondary collapse of the airways occurs. Future work could compare the novel results of the clinical scores presented in this paper against veterinarian-assessed clinical signs and/or other objective measures of these traits. However, a clear message from the current study is that specific closed questioning elicits very different responses than simply asking owners whether their dog has a problem or not, which may elicit wishful responses that ‘normalise’ away unpleasant thoughts of ill health in their dog.

### Owner perception of health problems

The health section of this study relatively reported high levels of disease, even though the study population was relatively young. Despite these high levels of acknowledged disease, the majority of owners in this population paradoxically perceived their dogs summarily to be in the ‘best health possible’ or ‘very good health’. Owners perceptions of *their own* dog’s health compared with the *rest of their breed* revealed a concerning trend towards owner overestimation of their own dog’s relative health. Across all three breeds, owners most commonly reported that their dog was ‘healthier than average for the breed’. When all breeds were considered together, just 6.8% of owners considered their dog ‘less’ or ‘much less’ healthy than average for their breed, and this phenomenon was consistent across all three breeds investigated. This deflection phenomenon may be driven by cognitive dissonance, where owners are aware of health problems in their dog’s breed, but find accepting these problems in their own dog as psychologically uncomfortable, instead deflecting the issues to other individuals. This may have led to the skewed distribution seen here, where a large proportion of owners, whose dogs by definition must be *less* healthy than average for their breed, overestimate their own dog’s health. The commonality across the three study breeds indicates this phenomenon is common to all brachycephalic dog owners and may be linked to deeper psychological conflict associated with owning and loving an individual of a breed with well-known health issues at a breed level.

### Expectations of brachycephalic ownership

There were two main areas of ownership that violated owner expectation: veterinary costs and exercise levels, with over one fifth of owners underestimating each of these. The substantial disease burden in brachycephalic breeds commonly necessitates high-cost surgical intervention, as demonstrated in this study. Poor planning for such predictable costs may result in undertreatment with consequent welfare impact. In a study of North American small animal veterinarians, 57% of respondents indicated that client economic limitations affected their ability to provide the desired care for their patients on a daily basis [42]. Lack of awareness of the financial implications of pet ownership extends beyond brachycephalic dogs; in a recent PDSA Animal Welfare Report, 69% of dog owners underestimated the monthly cost of owning a dog [43]. The full financial implications of owning a brachycephalic dog should be thoroughly discussed with, and considered by, prospective brachycephalic dog owners. This may result in potential owners either deciding against purchasing these breeds that carry high risk of major costs or may encourage appropriate financial planning (e.g. pet insurance) for those who remain committed to purchasing these breeds.

Owners also commonly underestimated the level of exercise their dog required. This may result from wide public perception that brachycephalic dogs require minimal activity, arising due to their restricted exercise tolerance (a core clinical feature of BOAS) [39, 44]. For those brachycephalic dogs either unaffected or only mildly affected by BOAS, exercise levels are therefore likely to be higher than anticipated, particularly in young dogs. Encouraging safe levels of exercise in brachycephalic dogs is important to promote fitness levels and avoid obesity, a risk factor for BOAS [6], and thus these breeds should not be marketed as having low exercise requirements as an inherent, and often appealing, breed attribute.

### The dog-owner bond

The MDORS scores reported in the current study indicate that brachycephalic dog owners generally form strong relationships with their dogs, with mean scores higher for all three sub-scales across all three breeds compared to other recent studies utilising the MDORS [45–47]. Breed itself has previously been identified as highly influential upon levels of owner attachment to their dog [48], and, even among the three similarly-brachycephalic breeds in the current study, some breed variation was also observed. Pug owners exhibited higher emotional closeness with their dog than French Bulldog owners, and lower perceived costs of ownership than both Bulldog and French Bulldog owners. Further research is required to explain these breed-associated differences, but it is possible that as yet unknown differences in owner personality [49] or dog personality [50] may exist between these breeds; factors which have previously been found to influence attachment. Although they are all severely brachycephalic, there are other substantial differences between the three breeds in the current study. The Pug has a lower average bodyweight that the other two: Kennel Club ideal weight: 6.3–8.1 kg Pug; 11–12.5kg French Bulldog; 23-25kg Bulldog [51]. Small body size in other breeds such as the Chihuahua has been considered a precipitant of caregiving behaviour from, and high levels of attachment from, owners, who may view and treat their dog as a child [48]. Indeed, the dog–human dyad is believed to involve attachment bonds similar to those that characterise human caregiver–infant relationships [52]. It is possible that this caregiver–infant relationship may be triggered by and enhanced in small dogs with neotenous features, such as the Pug. One reason suggested for the stronger parent-child like bonds that women can develop with companion animals is the helplessness and total dependence of these animals upon a human caregiver, making them effectively ‘perpetual children’ which fulfil some women’s desire to nurture and care for living beings [53]. It is possible that some small brachycephalic dogs may fulfil this child-like role but further research is required to support this hypothesis.

Owner demographic factors were found to influence the dog-owner relationship in the present study. Owners without children in the household exhibited higher emotional closeness with their dog and higher levels of dog-owner interaction than owners with children in the household. This may be due to time constraints of parenting not allowing for the same level of dog-owner interactions afforded to owners without children, and the emotional demands of parenthood not allowing for the same level of emotional bonding with their dog. Indeed, in a qualitative study of female pet owners, individuals showed stronger attachments to pets that either preceded the birth of their children, or followed their children leaving home [54]. In the current study, female owners exhibited higher emotional closeness than male owners in agreement with several previous studies that also reported stronger attachments to pets in women than men [55–57]. It should be noted that around 90% of study participants were female in the current study, as is endemic in pet owner research, and as such further investigation of male brachycephalic dog owners is needed to draw deeper conclusions regarding this group.

Canine health status was associated with the perceived costs and dog-owner interactions subscales of the MDORS in the current study. Dogs with a worse health rating than the ‘best health possible’ reported higher perceived costs of ownership, and lower levels of dog-owner interaction. Owning a dog with health issues may invoke more daily caring responsibilities (e.g. cleaning skin folds), and lead to changes of plan to accommodate their dog (e.g. not spending time outdoors on hot days to avoid heatstroke) which would influence the MDORS perceived costs score, and may inhibit an owner’s ability to engage in certain activities (e.g. playing games with their dog, taking their dog in the car or to visit people), reducing the MDORS dog-owner interaction score.

Finally, expectations of ownership were associated with various elements of the dog-owner relationship, most prominently how well their dog behaved compared to prior expectation. Owners of dogs that behaved worse than expected exhibited reduced emotional closeness to their dog, higher perceived costs of ownership, and lower levels of dog-owner interaction. There appears to be a complex relationship between dog behaviour and the dog-owner relationship. Previous studies have found that owners whose dogs’ actual behaviour was farther from their ‘ideal’ behaviour were less strongly attached owners [52], as seen here, and owners who perceived their dog to have a fear-related behaviour problem perceived higher costs of ownership [46]. However, other studies have found that owners of dogs with higher levels of social fear/aggression (as assessed practically through standardised tests) had closer emotional bonds with their dog [46]. Dog behaviour itself was not measured in this study and thus conclusions cannot be drawn between actual behaviour and the dog-owner bond; however, owner expectations of behaviour may contribute substantially to the decision-making process about which breed to acquire at the outset of the ownership journey, and if not met, may ultimately play a role in relinquishment that terminates this journey. Perceptions that brachycephalic dogs are good with children and make good companions have previously been reported to positively influence breed selection [15]. However, veterinary clinical data from a recent UK study of French Bulldogs identified that aggression was the 13^th^ most commonly recorded disorder in that breed [28], which challenges beliefs about their ideal temperament which is described as ‘deeply affectionate’ in the UK breed standard [51] [58]. Ensuring that owners have realistic expectations of dog behaviour prior to acquisition and awareness that environment as well as genetics play important roles in developing positive behavioural traits is a priority.

Limitations of this study include the sampling strategy used and resulting study population. As described above, the sample was biased towards young brachycephalic dogs which may reflect both current trends in ownership, and the study requesting owners to complete the survey for their most recently acquired dog. As such, the resulting sample may not reflect the experiences of all brachycephalic dogs and their owners. In addition, the use of a convenience sample may bias this population to owners with certain beliefs or demographic characteristics, and the use of data from dogs and owners from different countries may complicate the generalisability of these results. Finally, the treatment of the MDORS scores as numeric linear outcome variables in statistical models, although standard practice within canine science to date, is not the gold standard way to handle fundamentally ordinal data.

## Conclusions

Ownership of brachycephalic dog breeds is a complex phenomenon, characterised by extremely strong dog-owner relationships and unrealistic perceptions of good health set against high levels of disease in relatively young dogs. Perceptual errors in owner beliefs appear to exist between their perspective of their own dog’s health versus the health of the rest of their breed, which may be fuelled by cognitive dissonance processes. Greater understanding of the biological underpinnings of the strong owner-dog relationships in brachycephalic breeds (e.g. via analysis of neurophysiological markers) and more detailed understanding of the emotional benefits of such breeds as companion animals (e.g. via qualitative studies) may further elucidate the international popularity of brachycephalic breeds.

## Supporting information

S1 File Additional statistical information. Generalised linear models of MDORS sub-scales: final models, full factorial models, and summary data from MDORS sub-component questions
(DOCX)

## References

[1] Honey L. Future health and welfare crises predicted for the brachycephalic dog population. Veterinary Record. 2017;181(21):550 10.1136/vr.j5429 29175912

[2] Kennel Club T. Breed Registration Statistics 2018. Available from: https://www.thekennelclub.org.uk/registration/breed-registration-statistics.

[3] Teng KT, McGreevy PD, Toribio J-ALML, Dhand NK. Trends in popularity of some morphological traits of purebred dogs in Australia. Canine Genetics and Epidemiology. 2016;3(1):2 10.1186/s40575-016-0032-2 PMC4820977 27051522

[4] American Kennel Club. Most Popular Dog Breeds–Full Ranking List 2018. Available from: https://www.akc.org/expert-advice/news/most-popular-dog-breeds-full-ranking-list/.

[5] O'Neill DG, Jackson C, Guy JH, Church DB, McGreevy PD, Thomson PC, et al Epidemiological associations between brachycephaly and upper respiratory tract disorders in dogs attending veterinary practices in England. Canine Genet Epidemiol. 2015;2 10.1186/s40575-015-0023-8 26401338 PMC4579368

[6] Packer RMA, Hendricks A, Tivers MS, Burn CC. Impact of Facial Conformation on Canine Health: Brachycephalic Obstructive Airway Syndrome. PLOS ONE. 2015;10(10):e0137496 10.1371/journal.pone.0137496 26509577 PMC4624979

[7] Packer RMA, Hendricks A, Burn CC. Impact of Facial Conformation on Canine Health: Corneal Ulceration. PLOS ONE. 2015;10(5):e0123827 10.1371/journal.pone.0123827 25969983 PMC4430292

[8] O’Neill DG, Lee MM, Brodbelt DC, Church DB, Sanchez RF. Corneal ulcerative disease in dogs under primary veterinary care in England: epidemiology and clinical management. Canine Genetics and Epidemiology. 2017;4(1):5 10.1186/s40575-017-0045-5 28630713 PMC5471714

[9] O'Neill DG, O'Sullivan AM, Manson EA, Church DB, Boag AK, McGreevy PD, et al Canine dystocia in 50 UK first-opinion emergency-care veterinary practices: prevalence and risk factors. Veterinary Record. 2017;181(4). 10.1136/vr.104108 28526775

[10] Ryan R, Gutierrez-Quintana R, ter Haar G, De Decker S. Prevalence of thoracic vertebral malformations in French bulldogs, Pugs and English bulldogs with and without associated neurological deficits. The Veterinary Journal. 2017;221:25–9. 10.1016/j.tvjl.2017.01.018 28283076

[11] Nationwide. Brachycephalic Breed Disease Prevalence Study 2017 [cited 2017 August]. Available from: http://nationwidedvm.com/wp-content/uploads/2017/03/NWBrachycelphalicStudy0317.pdf.

[12] Farrow T, Keown AJ, Farnworth MJ. An exploration of attitudes towards pedigree dogs and their disorders as expressed by a sample of companion animal veterinarians in New Zealand. N Z Vet J. 2014;62 10.1080/00480169.2014.902340 24624976

[13] King T, Marston LC, Bennett PC. Describing the ideal Australian companion dog. Applied Animal Behaviour Science. 2009;120(1):84–93. 10.1016/j.applanim.2009.04.011.

[14] Diverio S, Boccini B, Menchetti L, Bennett PC. The Italian perception of the ideal companion dog. Journal of Veterinary Behavior. 2016;12:27–35. 10.1016/j.jveb.2016.02.004.

[15] Packer RMA, Murphy D, Farnworth MJ. Purchasing popular purebreds: investigating the influence of breed-type on the pre-purchase motivations and behaviour of dog owners. Animal Welfare. 2017;26(2):191–201. 10.7120/09627286.26.2.191

[16] Ghirlanda S, Acerbi A, Herzog H, Serpell JA. Fashion vs. function in cultural evolution: the case of dog breed popularity. PLoS One. 2013;8 10.1371/journal.pone.0074770 24040341 PMC3770587

[17] Sandøe P, Kondrup SV, Bennett PC, Forkman B, Meyer I, Proschowsky HF, et al Why do people buy dogs with potential welfare problems related to extreme conformation and inherited disease? A representative study of Danish owners of four small dog breeds. PLOS ONE. 2017;12(2):e0172091 10.1371/journal.pone.0172091 28234931 PMC5325474

[18] Lorenz K, editor. Part and parcel in animal and human societies. Cambridge, MA: Harvard University Press; 1971.

[19] Archer J, Monton S. Preferences for Infant Facial Features in Pet Dogs and Cats. Ethology. 2011;117(3):217–26. 10.1111/j.1439-0310.2010.01863.x

[20] Packer RMA, Hendricks A, Burn CC. Do dog owners perceive the clinical signs related to conformational inherited disorders as ‘normal’ for the breed? A potential constraint to improving canine welfare. Anim Welfare. 2012;21 10.7120/096272812x13345905673809

[21] Lue TW, Pantenburg DP, Crawford PM. Impact of the owner-pet and client-veterinarian bond on the care that pets receive. Journal of the American Veterinary Medical Association. 2008;232(4):531–40. 10.2460/javma.232.4.531 18279086

[22] O'Neill DG, Jackson C, Guy JH, Church DB, McGreevy PD, Thomson PC, et al Epidemiological associations between brachycephaly and upper respiratory tract disorders in dogs attending veterinary practices in England. Canine Genetics and Epidemiology. 2015;2(1):10 Epub 14 July. 10.1186/s40575-015-0023-8 26401338 PMC4579368

[23] Kaye BM, Rutherford L, Perridge DJ, Ter Haar G. Relationship between brachycephalic airway syndrome and gastrointestinal signs in three breeds of dog. Journal of Small Animal Practice. 2018;59(11):670–3. 10.1111/jsap.12914 30094894

[24] The Kennel Club. Breed registration statistics: The Kennel Club Limited; 2018 [cited 2018 September 16]. Available from: http://www.thekennelclub.org.uk/registration/breed-registration-statistics/.

[25] Garbinsky E, Morewedge C, Shiv B. Interference of the End: Why Recency Bias in Memory Determines When a Food Is Consumed Again. Psychological Science. 2014;25(7):1466–74. 10.1177/0956797614534268 24894582

[26] Rockenbauer M, Olsen J, Czeizel A, Pedersen L, Sørensen H. Recall bias in a case-control surveillance system on the use of medicine during pregnancy. Epidemiology. 2001;12(4):461–6. 11416783 10.1097/00001648-200107000-00017

[27] Dwyer F, Bennett PC, Coleman GJ. Development of the Monash Dog Owner Relationship Scale (MDORS). Anthrozoös. 2006;19(3):243–56. 10.2752/089279306785415592

[28] O’Neill DG, Baral L, Church DB, Brodbelt DC, Packer RMA. Demography and disorders of the French Bulldog population under primary veterinary care in the UK in 2013. Canine Genetics and Epidemiology. 2018;5(1):3 10.1186/s40575-018-0057-9 29750111 PMC5932866

[29] O’Neill DG, Darwent EC, Church DB, Brodbelt DC. Demography and health of Pugs under primary veterinary care in England. Canine Genetics and Epidemiology. 2016;3(1):5 10.1186/s40575-016-0035-z 27293771 PMC4903005

[30] O'Neill D, Kadhim J, Church D, Brodbelt D, Skipper AM, Packer RMA. Demography and disorders of British Bulldogs under primary veterinary care in the UK in 2013. Canine Genet and Epidemiol. Submitted. 10.1186/s40575-018-0057-9 PMC5932866 29750111

[31] O’Neill DG, Jackson C, Guy JH, Church DB, McGreevy PD, Thomson PC, et al Epidemiological associations between brachycephaly and upper respiratory tract disorders in dogs attending veterinary practices in England. Canine Genetics and Epidemiology. 2015;2(1):10 10.1186/s40575-015-0023-8 26401338 PMC4579368

[32] O'Neill DG, O'Sullivan AM, Manson EA, Church DB, Boag AK, McGreevy PD, et al Canine dystocia in 50 UK first-opinion emergency-care veterinary practices: prevalence and risk factors. Veterinary Record. 2017;181(4):88-. 10.1136/vr.104108 28526775

[33] Veterinary Record News and Reports. Vets urged to report caesareans to the Kennel Club. Veterinary Record. 2010;167(23):885-. 10.1136/vr.c6843 21262666

[34] British Veterinary Association. Dog health and welfare: dog breeding and hereditary defects—reporting caesareans and surgical procedures. 2016. Available from: https://www.bva.co.uk/News-campaigns-and-policy/Policy/Companion-animals/Dog-health-and-welfare/#dog-breeding

[35] Davis MS, Cummings SL, Payton ME. Effect of brachycephaly and body condition score on respiratory thermoregulation of healthy dogs. Journal of the American Veterinary Medical Association. 2017;251(10):1160–5. 10.2460/javma.251.10.1160 29099251

[36] Bruchim Y, Klement E, Saragusty J, Finkeilstein E, Kass P, Aroch I. Heat Stroke in Dogs: A Retrospective Study of 54 Cases (1999–2004) and Analysis of Risk Factors for Death. Journal of Veterinary Internal Medicine. 2006;20(1):38–46. 10.1892/0891-6640(2006)20[38:hsidar]2.0.co;2 16496921

[37] Hemmelgarn C, Gannon K. Heatstroke: thermoregulation, pathophysiology, and predisposing factors. Compend Contin Educ Vet. 2013;35(7):E4 23677841

[38] Hendricks JC, Kline LR, Kovalski RJ, O'Brien JA, Morrison AR, Pack AI. The English bulldog: a natural model of sleep-disordered breathing. Journal of Applied Physiology. 1987;63(4):1344–50. 10.1152/jappl.1987.63.4.1344 . 3693167

[39] Roedler FS, Pohl S, Oechtering GU. How does severe brachycephaly affect dog’s lives? Results of a structured preoperative owner questionnaire. Vet J. 2013;198 10.1016/j.tvjl.2013.02.004 24176279

[40] Roedler FS, Pohl S, Oechtering GU. How does severe brachycephaly affect dog’s lives? Results of a structured preoperative owner questionnaire. The Veterinary Journal. 2013;198(3):606–10. 10.1016/j.tvjl.2013.09.009 24176279

[41] Poncet CM, Dupre GP, Freiche VG, Estrada MM, Poubanne YA, Bouvy BM. Prevalence of gastrointestinal tract lesions in 73 brachycephalic dogs with upper respiratory syndrome. Journal of Small Animal Practice. 2005;46(6):273–9. 10.1111/j.1748-5827.2005.tb00320.x 15971897

[42] Kipperman BS, Kass PH, Rishniw M. Factors that influence small animal veterinarians’ opinions and actions regarding cost of care and effects of economic limitations on patient care and outcome and professional career satisfaction and burnout. Journal of the American Veterinary Medical Association. 2017;250(7):785–94. 10.2460/javma.250.7.785 28306486

[43] PDSA. PDSA Animal Wellbeing Report 2018 2018. Available from: https://www.pdsa.org.uk/media/4371/paw-2018-full-web-ready.pdf.

[44] Lilja-Maula L, Lappalainen AK, Hyytiäinen HK, Kuusela E, Kaimio M, Schildt K, et al Comparison of submaximal exercise test results and severity of brachycephalic obstructive airway syndrome in English bulldogs. The Veterinary Journal. 2017;219:22–6. 10.1016/j.tvjl.2016.11.019 28093105

[45] Packer RMA, Volk HA, Fowkes RC. Physiological reactivity to spontaneously occurring seizure activity in dogs with epilepsy and their carers. Physiology & Behavior. 2017;177:27–33. 10.1016/j.physbeh.2017.04.008. 28412282

[46] Meyer I, Forkman B. Dog and owner characteristics affecting the dog–owner relationship. Journal of Veterinary Behavior. 2014;9(4):143–50. 10.1016/j.jveb.2014.03.002.

[47] Rohlf VI, Bennett PC, Toukhsati S, Coleman G. Beliefs Underlying Dog Owners' Health Care Behaviors: Results from a Large, Self-Selected, Internet Sample. Anthrozoös. 2012;25(2):171–85. 10.2752/175303712X13316289505341

[48] Sandøe P, Kondrup SV, Bennett PC, Forkman B, Meyer I, Proschowsky HF. Why do people buy dogs with potential welfare problems related to extreme conformation and inherited disease? A representative study of Danish owners of four small dog breeds. PLoS One. 2017;12 10.1371/journal.pone.0172091 28234931 PMC5325474

[49] El-Alayli A, Lystad AL, Webb SR, Hollingsworth SL, Ciolli JL. Reigning Cats and Dogs: A Pet-Enhancement Bias and Its Link to Pet Attachment, Pet-Self Similarity, Self-Enhancement, and Well-Being. Basic and Applied Social Psychology. 2006;28(2):131–43. 10.1207/s15324834basp2802_3

[50] Hoffman CL, Chen P, Serpell JA, Jacobson KC. Do Dog Behavioral Characteristics Predict the Quality of the Relationship between Dogs and Their Owners? Human-animal interaction bulletin. 2013;1(1):20–37. . 25685855 PMC4326091

[51] The Kennel Club. Breed Standards Information: Dog Breeds & Groups: The Kennel Club,; 2018 [cited 2018 April 22]. Available from: https://www.thekennelclub.org.uk/activities/dog-showing/breed-standards/.

[52] Serpell J. Evidence for an association between pet behavior and owner attachment levels. Appl Anim Behav Sci. 1996;47(1–2):49–60.

[53] Margolies L. The long good-bye: Women, companion animals, and maternal loss. Clinical Social Work Journal. 1999;27(3):289–304.

[54] Turner WG. Our new children: The surrogate role of companion animals in women's lives. The Qualitative Report. 2001;6(1):1–10.

[55] Johnson T, Garrity T, Stallones L. Psychometric evaluation of the Lexington Attachment to Pets Scale. Anthrozoos. 1992;5(3):160–75.

[56] Kidd A, Kidd R. Factors in adults attitudes toward pets. Psychological Reports. 1989;65(3):903–10. 10.2466/pr0.1990.66.3.775 2377695

[57] Woodward L, Bauer A. People and their pets: A relational perspective on interpersonal complementarity and attachment in companion animal owners. Society & Animals. 2007;15(2):169–89.

[58] Kennel Club T. French Bulldog Breed Standard. Available from: https://www.thekennelclub.org.uk/services/public/breed/standard.aspx?id=4088.

