# Supplementary material for: Great expectations, inconvenient truths, and the paradoxes of the dog-owner relationship for owners of brachycephalic dogs
Source: PLoS One. 2019 Jul 19;14(7):e0219918. doi: 10.1371/journal.pone.0219918 (PMC6641206; doi:10.1371/journal.pone.0219918)
Supplement: S1 File — Generalised linear models of MDORS sub-scales: final models, full factorial models, and summary data from MDORS sub-component questions (DOCX) [file pone.0219918.s001.docx]

**Table S1. Final model 1 - Generalised linear mixed model of predictors of MDORS sub-scale Dog-Owner Interactions in a population of brachycephalic breeds (Pugs, Bulldogs and French Bulldogs).**

| **Variable** | **Sub-category** | **Coefficient**  **(95% CI)** | **SE** | **t** | **P** |
| --- | --- | --- | --- | --- | --- |
| Intercept | - | 4.14 (3.53 – 4.75) | 0.31 | 13.36 | <0.001 |
| Dog age (months) | - | -0.01 (-0.03 – 0.02) | 0.00 | -8.35 | <0.001 |
| Children in household | No | 0.01 (0.06 – 0.13) | 0.02 | 5.27 | <0.001 |
|  | Yes | *Reference* | | | |
| Expectations met – maintenance levels | Less than expected | -0.09 (-0.15 – -0.02) | 0.03 | -2.70 | 0.007 |
|  | More than expected | 0.05 (-0.01 – 0.11) | 0.03 | 1.57 | 0.116 |
|  | Yes | *Reference* | | | |
| Expectations met – behaviour | Better than expected | 0.01 (-0.04 – 0.05) | 0.02 | 0.26 | 0.793 |
|  | Worse than expected | -0.07 (-0.12 – -0.15) | 0.03 | -2.49 | 0.013 |
|  | Yes | *Reference* | | | |
| Health rating | 1: Could not be worse | -0.12 (-0.48 – 0.24) | 0.18 | -0.65 | 0.514 |
|  | 2: Very poor health | -0.08 (-0.22 – 0.05) | 0.07 | -1.17 | 0.241 |
|  | 3: Moderately poor health | -0.13 (-0.22 – -0.05) | 0.04 | -0.31 | 0.002 |
|  | 4: Good health | -0.04 (-0.11 – 0.03) | 0.04 | -1.12 | 0.261 |
|  | 5: Moderately good health | -0.03 (-0.09 – 0.03) | 0.03 | -1.24 | 0.025 |
|  | 6: Very good health | -0.03 (-0.07 – 0.02) | 0.02 | -1.19 | 0.017 |
|  | 7: Best health possible | *Reference* | | | |

N.B. Owner country of origin is included as a random effect^1^

*All variables in final model were significant in the initial full model prior to backwards elimination, as demonstrated in Table S4

^1^AIC with random effect =2219.27, AIC without random effect=2237.82

**Table S2. Final model 2 - Generalised linear mixed model of predictors of MDORS sub-scale Perceived Emotional Closeness in a population of brachycephalic breeds (Pugs, Bulldogs and French Bulldogs).**

N.B. Owner country of origin is included as a random effect^1^

| **Variable** | **Sub-category** | **Coefficient**  **(95% CI)** | **SE** | **t** | **P** |
| --- | --- | --- | --- | --- | --- |
| Intercept | - | 4.18 (3.30 – 5.06) | 0.45 | 9.32 | <0.001 |
| Breed | Bulldog | -0.02 (-0.08 – 0.05) | 0.03 | -0.50 | 0.620 |
|  | French Bulldog | -0.10 (-0.16 – -0.04) | 0.03 | -3.20 | 0.001 |
|  | Pug | *Reference* | | | |
| Children in household | No | 0.10 (0.05 – 0.15) | 0.03 | 3.93 | <0.001 |
|  | Yes | *Reference* | | | |
| Owner Gender | Female | 0.13 (0.05 – 0.22) | 0.04 | 3.13 | 0.002 |
|  | Male | *Reference* | | | |
| Expectations met – behaviour | Better than expected | 0.04 (-0.02 – 0.11) | 0.03 | 1.35 | 0.178 |
|  | Worse than expected | -0.13 (-0.21 – -0.05) | 0.04 | -3.16 | 0.002 |
|  | Yes | *Reference* | | | |

*All variables in final model were significant in the initial full model prior to backwards elimination, as demonstrated in Table S5

^1^AIC with random effect =3750.09, AIC without random effect=3760.95

**Table S3. Final model 3 - Generalised linear mixed model of predictors of MDORS sub-scale Perceived Costs in a population of brachycephalic breeds (Pugs, Bulldogs and French Bulldogs).**

N.B. Owner country of origin is included as a random effect^1^

*All variables in final model were significant in the initial full model prior to backwards elimination, as demonstrated in Table S6

^1^AIC with random effect =3027.26, AIC without random effect=3037.09

| **Variable** | **Sub-category** | **Coefficient**  **(95% CI)** | **SE** | **t** | **P** | |
| --- | --- | --- | --- | --- | --- | --- |
| Intercept | - | 4.33 (3.58 – 5.09) | 0.38 | 11.29 | <0.001 | |
| Breed | Bulldog | -0.11 (-0.16 – -0.05) | 0.03 | -3.71 | <0.001 | |
|  | French Bulldog | -0.07 (-0.12 – -0.02) | 0.03 | -2.56 | 0.011 | |
|  | Pug | *Reference* | | | | |
| First time owner | No | 0.20 (0.15 – 0.25) | 0.03 | 7.81 | <0.001 | |
|  | Yes | *Reference* | | | | |
| Expectations met – maintenance levels | Less than expected | 0.02 (-0.06 – 0.10) | 0.04 | 0.47 | 0.636 | |
|  | More than expected | -0.26 (-0.34 – -0.18) | 0.04 | -6.50 | <0.001 | |
|  | Yes | *Reference* | | | | |
| Expectations met – behaviour | Better than expected | 0.03 (-0.02 – 0.09) | 0.03 | 1.177 | 0.244 | |
|  | Worse than expected | -0.31 (-0.34 – -0.24) | 0.04 | -8.83 | <0.001 | |
|  | Yes | *Reference* | | | | |
| Expectations met – veterinary costs | Expected to pay less | -0.13 (-0.19 – -0.07) | 0.03 | -4.33 | | <0.001 |
|  | Expected to pay more | - 1. (-0.06 – 0.08) | 0.03 | 0.35 | | 0.726 |
|  | Yes | *Reference* | | | | |
| Health rating | 1: Could not be worse | -0.08 (-0.53 – 0.37) | 0.23 | -0.33 | <0.001 | |
|  | 2: Very poor health | -0.32 (-0.48 – -0.15) | 0.09 | -3.70 | <0.001 | |
|  | 3: Moderately poor health | -0.34 (-0.45 – -0.23) | 0.06 | -5.94 | <0.001 | |
|  | 4: Good health | -0.19 (-0.28 – -0.10) | 0.05 | -4.05 | <0.001 | |
|  | 5: Moderately good health | -0.16 (-0.23 – -0.08) | 0.04 | -4.09 | <0.001 | |
|  | 6: Very good health | -0.12 (-0.17 – -0.06) | 0.03 | -4.25 | <0.001 | |
|  | 7: Best health possible | *Reference* | | | | |

**Tables S4-6 demonstrate the initial full models of the final models presented in Table S1-3, including all twenty independent variables:**

1. Breed
2. Age
3. Owner age
4. Owner sex
5. Whether they were a first time dog owner (1/0)
6. Children in household (1/0)
7. Veterinary experiences (vet costs per year, £)
8. Veterinary experiences (vet costs to date, £)
9. Number of conformation-related surgeries
10. ORB
11. Heat intolerance score
12. Eating difficulty score
13. Sleeping dysfunction score
14. Number of perceived health problems
15. Health compared to the rest of their dog’s breed
16. Overall health rating
17. Expectations vs. Reality: veterinary costs
18. Expectations vs. Reality: exercise levels
19. Expectations vs. Reality: overall behaviour
20. Expectations vs. Reality: maintenance levels

**Table S4. Initial full factorial model 1 - GLMM of predictors of MDORS sub-scale Dog-Owner Interactions in a population of brachycephalic breeds (Pugs, Bulldogs and French Bulldogs).** Owner country of origin included as a random effect

| **Variable** | **Sub-category** | **Coefficient** | **SE** | **t** | **P** |
| --- | --- | --- | --- | --- | --- |
| Intercept | - | 4.06 | 0.30 | 13.98 | <0.001 |
| Breed | Bulldog | 0.04 | 0.02 | 1.73 | 0.083 |
|  | French Bulldog | 0.04 | 0.02 | 2.11 | 0.035 |
|  | Pug | *Reference* | | | |
| Dog age | - | -0.03 | 0.01 | -7.42 | <0.001 |
| Owner age (years) | 18-34 | 0.01 | 0.04 | 0.23 | 0.820 |
|  | 35-54 | 0.01 | 0.04 | 0.09 | 0.921 |
|  | 55-74 | *Reference* | | | |
| Owner sex | Female | 0.01 | 0.03 | 0.27 | 0.069 |
|  | Male | *Reference* | | | |
| First time owner | No | 0.01 | 0.02 | 0.46 | 0.647 |
|  | Yes | *Reference* | | | |
| Children in household | No | 0.07 | 0.02 | 3.75 | <0.001 |
|  | Yes | *Reference* | | | |
| Vet costs per year (£) | - | -0.00 | 0.00 | -1.80 | 0.072 |
| Vet costs to date (£) | - | 0.00 | 0.00 | 2.23 | 0.026 |
| # conformation-related surgeries | - | -0.01 | 0.01 | 0.48 | 0.629 |
| ORB | - | -0.01 | 0.01 | -0.65 | 0.518 |
| Heat intolerance score | - | -0.01 | 0.01 | -1.12 | 0.264 |
| Eating difficulty score | - | -0.01 | 0.01 | -1.23 | 0.218 |
| Sleeping dysfunction score | - | 0.01 | 0.01 | 5.07 | <0.001 |
| # of perceived health problems | - | 0.02 | 0.02 | 1.44 | 0.149 |
| Health compared with the rest of the breed | Much less healthy | 0.16 | 0.10 | 1.70 | 0.089 |
|  | Less healthy | 0.14 | 0.05 | 2.73 | 0.06 |
|  | Average for the breed | *Reference* | | | |
|  | Healthier | -0.03 | 0.02 | -1.12 | 0.262 |
|  | Much healthier | 0.16 | 0.10 | 1.70 | 0.089 |
| Expectations met – exercise | Less than expected | -0.03 | 0.03 | -0.75 | 0.452 |
|  | More than expected | 0.02 | 0.02 | 0.84 | 0.396 |
|  | Yes | *Reference* | | | |
| Expectations met – maintenance levels | Less than expected | -0.09 | 0.03 | -2.54 | 0.011 |
|  | More than expected | 0.06 | 0.03 | 1.85 | 0.064 |
|  | Yes | *Reference* | | | |
| Expectations met – behaviour | Better than expected | -0.01 | 0.02 | -0.07 | 0.94 |
|  | Worse than expected | -0.08 | 0.03 | -2.58 | 0.01 |
|  | Yes | *Reference* | | | |
| Expectations met – veterinary costs | Expected to pay less | -0.02 | 0.03 | -0.77 | 0.443 |
|  | Expected to pay more | -0.01 | 0.03 | -0.43 | 0.671 |
|  | Yes | *Reference* | | | |
| Health rating | 1: Could not be worse | -0.28 | 0.21 | -1.37 | 0.172 |
|  | 2: Very poor health | -0.22 | 0.09 | -2.36 | 0.018 |
|  | 3: Moderately poor health | -0.20 | 0.06 | -3.45 | 0.001 |
|  | 4: Good health | -0.04 | 0.04 | -0.99 | 0.322 |
|  | 5: Moderately good health | -0.06 | 0.04 | -1.59 | 0.111 |
|  | 6: Very good health | -0.01 | 0.02 | -0.28 | 0.778 |
|  | 7: Best health possible | *Reference* | | | |

**Table S5. Initial full factorial model 2 - GLMM of predictors of MDORS sub-scale Perceived Emotional Closeness in a population of brachycephalic breeds (Pugs, Bulldogs and French Bulldogs).** Owner country of origin included as a random effect

| **Variable** | **Sub-category** | **Coefficient** | **SE** | **t** | **P** |
| --- | --- | --- | --- | --- | --- |
| Intercept | - | 3.97 | 045 | 8.90 | <0.001 |
| Breed | Bulldog | -0.07 | 0.04 | -1.93 | 0.05 |
|  | French Bulldog | -0.11 | 0.03 | -0.35 | 0.001 |
|  | Pug | *Reference* | | | |
| Dog age | - | -0.01 | 0.01 | -1.62 | 0.107 |
| Owner age (years) | 18-34 | 0.24 | 0.06 | 4.17 | <0.001 |
|  | 35-54 | 0.09 | 0.06 | 1.60 | 0.109 |
|  | 55-74 | *Reference* | | | |
| Owner sex | Female | 0.12 | 0.05 | 2.60 | 0.009 |
|  | Male | *Reference* | | | |
| First time owner | No | 0.07 | 0.03 | 2.08 | 0.038 |
|  | Yes | *Reference* | | | |
| Children in household | No | 0.07 | 0.03 | 2.42 | 0.016 |
|  | Yes | *Reference* | | | |
| Vet costs per year (£) | - | -0.00 | 0.00 | -0.18 | 0.856 |
| Vet costs to date (£) | - | 0.00 | 0.00 | 1.79 | 0.073 |
| # conformation-related surgeries | - | 0.01 | 0.01 | 0.20 | 0.842 |
| ORB | - | 0.01 | 0.01 | 0.21 | 0.836 |
| Heat intolerance score | - | -0.01 | 0.01 | -1.20 | 0.230 |
| Eating difficulty score | - | -0.01 | 0.01 | -0.39 | 0.698 |
| Sleeping dysfunction score | - | 0.02 | 0.01 | 5.44 | <0.001 |
| # of perceived health problems | - | 0.01 | 0.02 | 0.39 | 0.694 |
| Health compared with the rest of the breed | Much less healthy | 0.16 | 0.15 | 1.12 | 0.254 |
|  | Less healthy | 0.09 | 0.08 | 1.14 | 2.54 |
|  | Average for the breed | *Reference* | | | |
|  | Healthier | 0.01 | 0.04 | 0.39 | 0.698 |
|  | Much healthier | 0.10 | 0.04 | 2.20 | 0.028 |
| Expectations met – exercise | Less than expected | 0.07 | 0.05 | 1.19 | 2.33 |
|  | More than expected | -0.08 | 0.03 | -2.24 | 0.025 |
|  | Yes | *Reference* | | | |
| Expectations met – maintenance levels | Less than expected | 0.08 | 0.05 | 1.53 | 0.126 |
|  | More than expected | -0.08 | 0.05 | -1.57 | 0.117 |
|  | Yes | *Reference* | | | |
| Expectations met – behaviour | Better than expected | 0.03 | 0.04 | 0.82 | 0.415 |
|  | Worse than expected | -0.17 | 0.04 | -3.91 | <0.001 |
|  | Yes | *Reference* | | | |
| Expectations met – veterinary costs | Expected to pay less | 0.06 | 0.04 | 1.44 | 0.149 |
|  | Expected to pay more | 0.03 | 0.04 | 0.65 | 0.519 |
|  | Yes | *Reference* | | | |
| Health rating | 1: Could not be worse | 0.03 | 0.31 | 0.09 | 0.931 |
|  | 2: Very poor health | -0.35 | 0.14 | -2.51 | 0.012 |
|  | 3: Moderately poor health | -0.16 | 0.09 | -1.87 | 0.062 |
|  | 4: Good health | -0.05 | 0.06 | -0.83 | 0.408 |
|  | 5: Moderately good health | -0.05 | 0.05 | -1.02 | 0.308 |
|  | 6: Very good health | -0.01 | 0.04 | -0.29 | 0.771 |
|  | 7: Best health possible | *Reference* | | | |

**Table S6. Initial full factorial model 3 - GLMM of predictors of MDORS sub-scale Perceived Costs in a population of brachycephalic breeds (Pugs, Bulldogs and French Bulldogs).** N.B. Owner country of origin included as a random effect

| **Variable** | **Sub-category** | **Coefficient** | **SE** | **t** | **P** |
| --- | --- | --- | --- | --- | --- |
| Intercept | - | 4.41 | 0.38 | 11.49 | <0.001 |
| Breed | Bulldog | -0.10 | 0.03 | -3.46 | 0.001 |
|  | French Bulldog | -0.04 | 0.03 | -1.28 | 0.200 |
|  | Pug | *Reference* | | | |
| Dog age | - | 0.01 | 0.01 | 1.29 | 0.196 |
| Owner age (years) | 18-34 | -0.07 | 0.05 | -1.33 | 0.183 |
|  | 35-54 | -0.03 | 0.05 | -0.52 | 0.603 |
|  | 55-74 | *Reference* | | | |
| Owner sex | Female | 0.08 | 0.04 | 1.96 | 0.051 |
|  | Male | *Reference* | | | |
| First time owner | No | 0.16 | 0.03 | 5.82 | <0.001 |
|  | Yes | *Reference* | | | |
| Children in household | No | -0.01 | 0.03 | -0.05 | 0.962 |
|  | Yes | *Reference* | | | |
| Vet costs per year (£) | - | 0.00 | 0.00 | 0.58 | 0.577 |
| Vet costs to date (£) | - | 0.00 | 0.00 | 0.56 | 0.563 |
| # conformation-related surgeries | - | 0.03 | 0.02 | 1.54 | 0.125 |
| ORB | - | -0.01 | 0.01 | -0.63 | 0.530 |
| Heat intolerance score | - | -0.02 | 0.01 | -2.09 | 0.037 |
| Eating difficulty score | - | -0.02 | 0.01 | -2.40 | 0.016 |
| Sleeping dysfunction score | - | 0.01 | 0.01 | 0.55 | 0.582 |
| # of perceived health problems | - | -0.04 | 0.02 | -2.01 | 0.044 |
| Health compared with the rest of the breed | Much less healthy | 0.04 | 0.12 | 0.29 | 0.772 |
|  | Less healthy | -0.06 | 0.07 | -0.97 | 0.332 |
|  | Average for the breed | *Reference* | | | |
|  | Healthier | -0.01 | 0.03 | -0.23 | 0.817 |
|  | Much healthier | -0.01 | 0.04 | -0.16 | 0.873 |
| Expectations met – exercise | Less than expected | 0.04 | 0.05 | 0.75 | 0.454 |
|  | More than expected | -0.02 | 0.03 | -0.73 | 0.465 |
|  | Yes | *Reference* | | | |
| Expectations met – maintenance levels | Less than expected | 0.04 | 0.04 | 0.93 | 0.354 |
|  | More than expected | -0.23 | 0.04 | -5.63 | <0.001 |
|  | Yes | *Reference* | | | |
| Expectations met – behaviour | Better than expected | 0.05 | 0.03 | 1.53 | 0.125 |
|  | Worse than expected | -0.29 | 0.04 | -7.78 | <0.001 |
|  | Yes | *Reference* | | | |
| Expectations met – veterinary costs | Expected to pay less | 0.02 | 0.04 | 0.45 | 0.654 |
|  | Expected to pay more | -0.14 | 0.03 | -4.16 | <0.001 |
|  | Yes | *Reference* | | | |
| Health rating | 1: Could not be worse | 0.05 | 0.26 | 0.20 | 0.842 |
|  | 2: Very poor health | -0.35 | 0.12 | -2.97 | 0.003 |
|  | 3: Moderately poor health | -0.32 | 0.08 | -4.20 | <0.001 |
|  | 4: Good health | -0.15 | 0.05 | -2.82 | 0.005 |
|  | 5: Moderately good health | -0.18 | 0.05 | -3.95 | <0.001 |
|  | 6: Very good health | -0.11 | 0.03 | -3.79 | <0.001 |
|  | 7: Best health possible | *Reference* | | | |

**Table S7. Summary data from MDORS sub-component questions**

| **MDORS sub-scale** | **Question** | **Median (IQR)** | **Score (%)** | | | | |
| --- | --- | --- | --- | --- | --- | --- | --- |
|  |  |  | **1** | **2** | **3** | **4** | **5** |
| Perceived Emotional Closeness  (PEC) | My dog gives me a reason to get up in the morning. | 5  (4-5) | 0.1 | 1.7 | 12.3 | 25.4 | 60.4 |
|  | How often do you tell your dog things you don’t tell anyone else? | 4  (1-5) | 27.0 | 5.9 | 11.4 | 15.6 | 40.1 |
|  | My dog helps me get through tough times. | 5  (4-5) | 0.3 | 1.4 | 10.7 | 26.7 | 60.9 |
|  | If everyone else left me, my dog would still be there for me. | 5  (4-5) | 0.3 | 1.1 | 7.2 | 25.6 | 65.9 |
|  | My dog provides me with constant companionship. | 5  (4-5) | 0.1 | 0.8 | 4.9 | 27.2 | 67.0 |
|  | My dog is there whenever I need to be comforted. | 4  (4-5) | 0.4 | 1.2 | 9.7 | 28.4 | 60.3 |
|  | I wish my dog and I never had to be apart | 4  (3-5) | 2.0 | 11.6 | 22.9 | 25.4 | 38.1 |
|  | My dog is constantly attentive to me. | 4  (3-5) | 0.7 | 6.9 | 18.2 | 36.4 | 37.8 |
|  | How traumatic do you think it will be for you when your dog dies? | 5  (5-5) | 0.0 | 0.1 | 1.2 | 16.0 | 82.7 |
|  | I would like to have my dog near me all the time. | 5  (4-5) | 0.2 | 2.7 | 11.5 | 30.7 | 54.9 |
| Perceived Costs (PC) | It is annoying that sometimes I have to change my plans because of my dog. | 4  (3-5) | 1.4 | 10.3 | 17.8 | 33.7 | 36.8 |
|  | How hard is it to look after your dog? | 4  (3-4) | 6.5 | 38.4 | 38.4 | 30.6 | 23.6 |
|  | How often do you feel that looking after your dog is a chore? | 5  (5-5) | 1.7 | 2.2 | 4.7 | 13.7 | 77.3 |
|  | How often does your dog stop you from doing things you want to? | 5  (4-5) | 1.1 | 2.3 | 6.6 | 27.9 | 61.8 |
|  | How often do you feel that having a dog is more trouble than it’s worth? | 5  (5-5) | 0.6 | 0.4 | 1.2 | 61. | 91.7 |
|  | My dog costs too much money. | 4  (3-5) | 2.4 | 7.4 | 20.6 | 31.9 | 37.7 |
|  | It bothers me that my dog stops me doing things I enjoyed before I owned it. | 5  (4-5) | 0.7 | 2.2 | 9.5 | 27.7 | 59.9 |
|  | There are major aspects of owning a dog I don’t like. | 4  (4-5) | 1.8 | 4.7 | 17.3 | 30.1 | 46.1 |
|  | My dog makes too much mess. | 4  (4-5) | 1.5 | 5.1 | 12.6 | 34.5 | 46.3 |
| Dog-Owner Interactions | How often do you hug your dog? | 5  (5-5) | 1.2 | 0.0 | 0.6 | 3.7 | 94.5 |
|  | How often do you kiss your dog? | 5  (5-5) | 4.6 | 0.8 | 2.0 | 6.4 | 86.3 |
|  | How often do you groom your dog? | 4  (3-4) | 1.7 | 17.7 | 27.9 | 29.6 | 23.3 |
|  | How often do you have your dog with you while relaxing, i.e. watching TV? | 5  (5-5) | 0.2 | 0.3 | 0.1 | 1.9 | 97.5 |
|  | How often do you play games with your dog? | 5  (5-5) | 0.6 | 0.8 | 3.0 | 13.1 | 82.5 |
|  | How often do you give your dog food treats? | 5  (4-5) | 1.9 | 1.9 | 7.2 | 19.4 | 69.5 |
|  | How often do you take your dog in the car? | 4  (3-4) | 2.5 | 15.3 | 22.5 | 37.0 | 22.7 |
|  | How often do you buy your dog presents? | 3  (2-3) | 3.3 | 41.6 | 36.1 | 15.2 | 3.8 |
|  | How often do you take your dog to visit people? | 3  (2-4) | 7.0 | 20.8 | 29.4 | 31.4 | 11.3 |
